# Stability and dermatological safety assessment of *Cryptocarya alba* (Peumo) and *Laureliopsis philippiana* (Tepa) essential oil-carrying dry shampoo formulations for dermal use in dogs

**DOI:** 10.3389/fvets.2026.1918882

**Published:** 2026-10-05

**Authors:** Martín Varas, Martina Jacobs, Nicole Rojas, Paola Ramos, Flavia Bruna, Gabriela Valenzuela-Barra, Olosmira Correa, Antonia Díaz-Parra, Manuel Ahumada, Gabriela Maturana, Irene Martínez, Francisco Abusleme, Belén Rivera, María Olga Bargsted, Daniela Siel, Jessica Bravo

**Affiliations:** 1 Laboratory of Bioactive Natural Products, School of Medicine, Center for Biomedical Research, Diego Portales University, Santiago, Chile; 2 Department of Chemical Engineering, Biotechnology and Materials, Faculty of Physical and Mathematical Sciences, University of Chile, Santiago, Chile; 3 Hormone and Cancer Biology Laboratory, Institute of Experimental Medicine and Biology of Cuyo (IMBECU), CONICET CCT Mendoza—National University of Cuyo (UNCuyo), Mendoza, Argentina; 4 Center of Odontological Research (CIO) From the Faculty of Odontology, National University of Cuyo (UNCuyo), Mendoza, Argentina; 5 Department of Pharmaceutical Sciences and Technology, Faculty of Chemical and Pharmaceutical Sciences, University of Chile, Santiago, Chile; 6 School of Biotechnology, Faculty of Sciences, Engineering and Technology, Mayor University, Santiago, Chile; 7 School of Veterinary Medicine, Faculty of Medicine and Health Sciences, Mayor University, Santiago, Chile; 8 Oftaderm Veterinary Clinic, Santiago, Chile; 9 Center for Biomedicine, Mayor University, Santiago, Chile

**Keywords:** essential oils, *Cryptocarya alba*, *Laureliopsis philippiana*, dry shampoo, canine superficial pyoderma, *Staphylococcus pseudintermedius*

## Abstract

**Introduction:**

Antibiotic resistance is a major concern in human and veterinary medicine, supporting the development of alternative topical products for companion animals. This study aimed to develop and evaluate essential oil (EO)-based dry shampoo formulations incorporating EOs from *Cryptocarya alba* and *Laureliopsis philippiana* as potential veterinary dermocosmetic platforms for canine skin care and complementary management of superficial pyoderma.

**Methods:**

Starch-based dry shampoo prototypes containing *C. alba* or *L. philippiana* EOs were developed using corn starch, tapioca starch, or their mixtures. Magnesium stearate (MgS) was evaluated as a functional excipient, and two mixing protocols were compared. Optimized formulations were subjected to physicochemical characterization, thermal, mechanical, and 9-month stability assessments, and in vitro antibacterial testing against *Staphylococcus pseudintermedius*. Preclinical dermal safety was evaluated in BALB/c mice through macroscopic irritation scoring, body weight monitoring, and histological analysis. Subsequently, an exploratory, randomized, controlled, blinded, four-arm parallel-group study was conducted in 32 client-owned dogs with superficial pyoderma allocated to receive SLP, SCA, vehicle shampoo, or a chlorhexidine-based reference treatment for 21 days.

**Results:**

MgS improved formulation homogeneity, fluidity, and reduced agglomeration, while the selected mixing protocol enhanced overall powder performance. The optimized formulations showed preliminary physical stability, although progressive changes in texture, fluidity, agglomeration tendency, and aroma were observed during storage. In vitro antibacterial activity was assay-dependent: no measurable inhibition zones were observed by agar diffusion, whereas serial dilution and viable-count assays showed concentration-dependent reductions in bacterial recovery, including effects associated with the vehicle formulation. No macroscopic or histological signs of dermal irritation were observed in mice. In dogs, the formulations were well tolerated under the evaluated conditions. Microbiological outcomes differed numerically among groups but not significantly; among the EO-containing formulations, SCA showed the most favorable exploratory profile, including culture negativity in 3/8 dogs and the highest proportion of dogs reaching a non-significant bacterial burden.

**Discussion:**

These findings support the feasibility of incorporating *C. alba* and *L. philippiana* EOs into starch-based dry shampoo prototypes and provide preliminary evidence of physical stability and dermal tolerability. However, the assay-dependent antibacterial response, alkaline formulation pH, limited batch replication, and exploratory clinical design preclude conclusions regarding clinical efficacy. Further studies should include formulation optimization, quantitative chemical stability assessment, dermal permeation studies, and larger randomized controlled clinical trials.

## 1 Introduction

Pyoderma is among the most common health problems in dogs and is often caused by bacteria, with *Staphylococcus pseudintermedius* being the most frequent agent (1). The traditional treatment for pyoderma includes antibiotics (2), but antibiotic misuse is now one of the main causes of bacterial resistance, a major concern for human and animal health. New antibiotic agents are urgently needed to treat multidrug-resistant bacteria (3). Topical pharmaceutical formulations are recommended for treatment of superficial pyoderma even in the presence of methicillin-resistant strains (2). Previous studies have explored alternative topical strategies for canine superficial pyoderma. For example, cyclodextrin-based protocols have shown efficacy comparable to systemic antibiotic therapy in selected cases, highlighting the potential of locally applied formulations to reduce reliance on systemic antimicrobials (4). However, the performance of topical therapies depends on multiple formulation-related factors, including the concentration of the active compound, surfactant use, pH, application frequency, and contact time (5, 6). Two to three applications per week with contact times between 5 and 10 min are recommended, but studies have shown that long-term treatments may disrupt the normal protective function of the dermis, which is especially relevant in patients with previously inflamed skin (6).

Plant EOs are complex mixtures containing terpenes, terpenoids, and phenyl terpenes, among other bioactive compounds (7, 8). Plants synthesize these secondary metabolites as a defense mechanism against pathogens, and due to their antioxidant, anti-inflammatory, antibacterial, and antifungal properties (7–10), EOs have been studied for their application in the pharmaceutical industry (11).

*Cryptocarya alba* (Peumo) and *Laureliopsis philippiana* (Tepa) are two tree species native to Chile that the Mapuche people have used to treat liver diseases, rheumatism, and wounds (12, 13). The EOs obtained from *C. alba* and *L. philippiana* are rich in 1,8-cineole, also known as eucalyptol, linalool, and safrole, among other compounds (13, 14), and their antibacterial activity has been reported against *Staphylococcus aureus*, *Escherichia coli*, *Pseudomonas aeruginosa*, and *Klebsiella pneumoniae*, among others (13, 14).

For veterinary topical formulations intended for repeated application, *in vivo* assessment is essential to establish both safety and biological performance before clinical use. Murine models are widely used to evaluate local dermal tolerance, including erythema, edema, histological alterations, and potential systemic effects, whereas studies in dogs, as the target species, allow preliminary evaluation of clinical and microbiological responses under real dermatological conditions (15–22).

This study hypothesized that essential oils from *C. alba* and *L. philippiana* could be incorporated into a starch-based dry shampoo with acceptable physical stability and local dermal tolerability, and that the resulting formulations could produce measurable microbiological responses in dogs with superficial pyoderma. Therefore, the main objective was to develop and characterize EO-loaded dry shampoo prototypes, assess their preclinical dermal safety, and evaluate their microbiological performance in client-owned dogs with superficial pyoderma.

## 2 Materials and methods

The experimental workflow of the study is summarized in Figure 1. Briefly, the methodology comprised five sequential stages: plant sampling and essential oil extraction, dry shampoo formulation development, physicochemical characterization, preclinical safety testing in mice, and clinical evaluation in dogs. Each stage was designed to progressively assess formulation feasibility, stability, safety, and microbiological performance.

**Figure 1:**
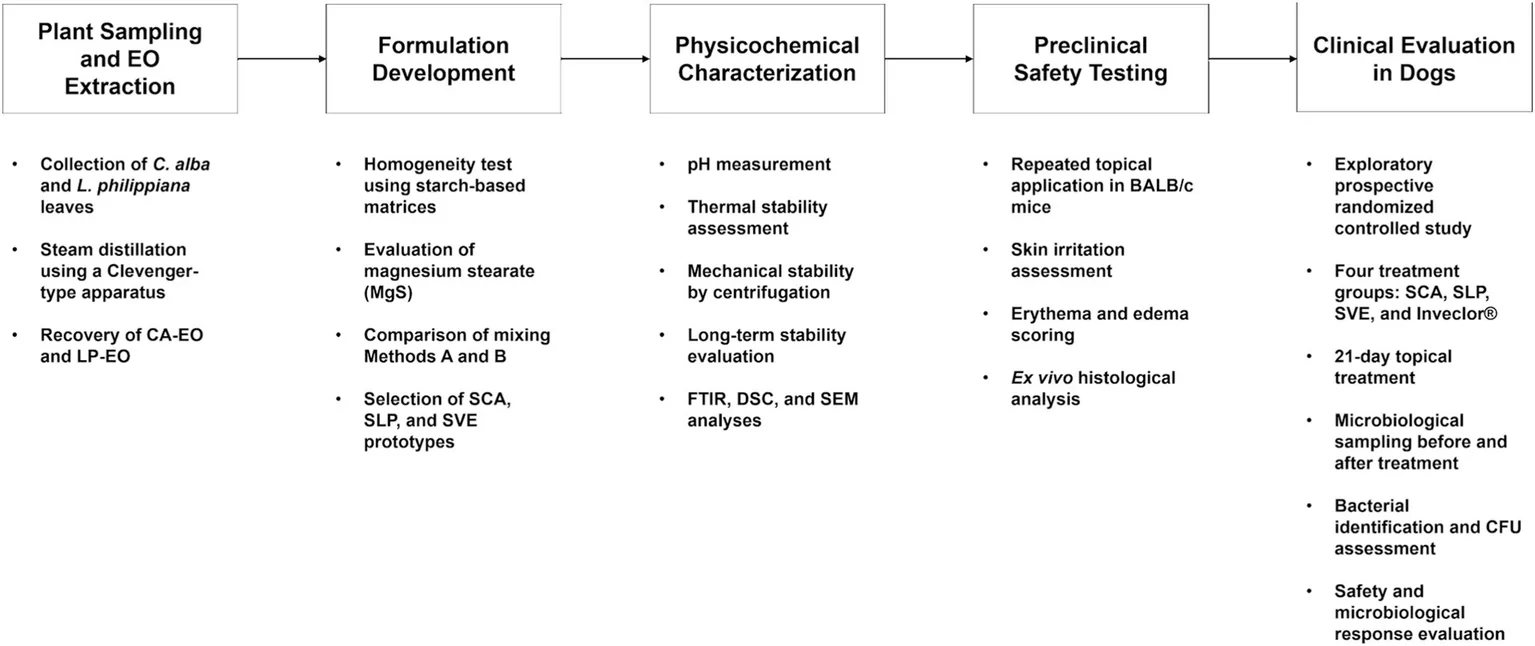
Experimental workflow of the study. The methodological sequence included plant sampling and essential oil (EO) extraction, formulation development, physicochemical characterization, preclinical safety testing in BALB/c mice, and clinical evaluation in dogs. CA-EO: *Cryptocarya alba* essential oil; LP-EO: *Laureliopsis philippiana* essential oil; MgS: magnesium stearate; SCA: dry shampoo containing *C. alba* essential oil; SLP: dry shampoo containing *L. philippiana* essential oil; SVE: vehicle formulation; FTIR: Fourier-transform infrared spectroscopy; DSC: differential scanning calorimetry; SEM: scanning electron microscopy; CFU: colony-forming units.

### 2.1 Sampling and extraction

Fresh leaves of *C. alba* (CA) were collected during spring (November 2024) at the end of the flowering period and before fruit formation, in Chicauma, Lampa, Metropolitan Region, Chile (33°12′3.8808″S, 70°58′8.1156″W). Similarly, fresh leaves of *L. philippiana* (LP) were collected in October 2024 during the anthesis period in Los Pellines, Valdivia, Los Ríos Region, Chile (39°44′42.0″S, 73°21′25.2″O). Voucher specimens were prepared for both species. CA (SQF 22901) was deposited in the Herbarium of the Faculty of Chemistry and Pharmacy, University of Chile, while LP (VALD 3560) was deposited in the Herbarium of the Universidad Austral de Chile. Plant material was processed for extraction on the day of collection or within 24 h thereafter.

EO extraction from both species was carried out immediately after collecting using a 10-L Clevenger-type apparatus via steam distillation. The process was conducted in batches until the entire plant material was distilled (1 h per batch in both cases). The resulting heterogeneous mixture was separated using a separatory funnel to recover the EO. The hydrosol was discarded. Thus, two EO were obtained: CA essential oil (CA-EO) and LP essential oil (LP-EO).

The chemical profile of these essential oils has been previously characterized by gas chromatography–mass spectrometry (GC–MS) by Jacobs *et al*. (23). In that analysis, a total of 62 volatile compounds were detected across both oils. CA-EO showed a more complex profile, with 43 identified compounds, among which sabinene and eucalyptol were the major constituents, representing 13.95 and 12.05% of the total chromatographic area, respectively. In contrast, LP-EO presented 20 identified compounds, with linalool, eucalyptol, and safrole as the predominant constituents, accounting for 37.91, 24.54, and 11.42% of the total chromatographic area, respectively. These findings confirm that CA-EO and LP-EO exhibit clearly distinct phytochemical profiles, which may contribute to differences in their biological and antibacterial properties.

The *in vitro* antibacterial activity of CA-EO and LP-EO against *Staphylococcus pseudintermedius* has also been previously reported by Jacobs et al. (23). In that study, both essential oils were evaluated against 12 *S. pseudintermedius* isolates from dogs with pyoderma, together with the reference strain *S. pseudintermedius* ATCC 49444, using agar diffusion and minimum inhibitory concentration (MIC) assays. Both oils showed moderate antibacterial activity, with inhibition zones ranging from 4.92 to 10.80 mm. CA-EO showed a higher mean inhibition diameter than LP-EO, with average values of 9.09 mm and 6.76 mm, respectively, corresponding to 37 and 28% of the activity observed for the reference antiseptic chlorhexidine. The most susceptible strains were SP9 for CA-EO and SP13 for LP-EO, with inhibition zones of 10.38 and 10.80 mm, respectively. MIC values were generally ≥4.096 mg mL^−1^ for both oils, except for LP-EO against strain SP12, which showed an MIC of 2.048 mg mL^−1^. These results support the antibacterial potential of both essential oils against *S. pseudintermedius*, although with lower activity than chlorhexidine.

### 2.2 Formulation and homogeneity test

To evaluate homogeneity, six shampoo formulations carrying LP-EO (SLP) were prepared in 10 g samples designed considering commercial dry shampoo formulation examples from AGRANA STARCH^1^ and AkzoNobel Surface Chemistry.^2^

To evaluate homogeneity, six shampoo formulations carrying LP-EO (SLP) were prepared in 10 g samples based on commercial products (AGRANA – MAISITA 9040 and Zephyrous Dry Shampoo 2,906–100) and taking as reference previous work on dry herbal powder shampoo formulations (24). Three starches were tested: cornstarch, tapioca, and a mixture of both. Magnesium stearate was selected because of its established use in solid pharmaceutical and cosmetic formulations and its reported influence on powder flow, particle cohesion, and agglomeration. These properties were considered particularly relevant for improving the incorporation and distribution of the lipophilic EO within the starch-based matrix. To evaluate its functional contribution, otherwise comparable formulations were prepared with and without MgS (25–27). Formulations were grouped according to the starch used, and each of these groups was subdivided into two, depending on the addition of MgS, resulting in the 6 formulations: formulation based on cornstarch without MgS (SLP1) and with MgS (SLP1-MgS), formulation based on tapioca without MgS (SLP2), and with MgS (SLP2-MgS), formulation based on a mixture of cornstarch and tapioca without MgS (SLP3), and with MgS (SLP3-MgS). For visual assessment of EO dispersion, LP-EO was previously mixed with Sudan Red B (Merck, Chile), an oil-soluble dye.

The starch was combined with MgS when applicable, followed by the addition of Sudan Red B–colored LP-EO. The mixture was agitated in an Incu-Shaker™ Mini at 200 rpm for 18 h, ground with a mortar and pestle to break agglomerates. Sodium lauryl ether sulfate (SLES) was subsequently incorporated as an anionic surfactant to improve the wetting and dispersion of the hydrophobic EO within the powder matrix and to provide a cleansing function consistent with the intended use of the formulation as a topical dry shampoo (28). The mixture was then agitated for an additional 2 h (Figure 2). This mixing method will be referred to as Method A. The resulting powders were evaluated using a qualitative scale based on seven organoleptic and physical parameters (Table 1), including homogeneity, texture, color, fluidity, and odor, scored on a 1 to 5 qualitative scale designed according to general organoleptic and physical evaluation criteria described for pharmaceutical and cosmetic formulations (28, 29). Samples with better visual uniformity and lower clumping tendency were selected for the mixing test.

**Figure 2:**
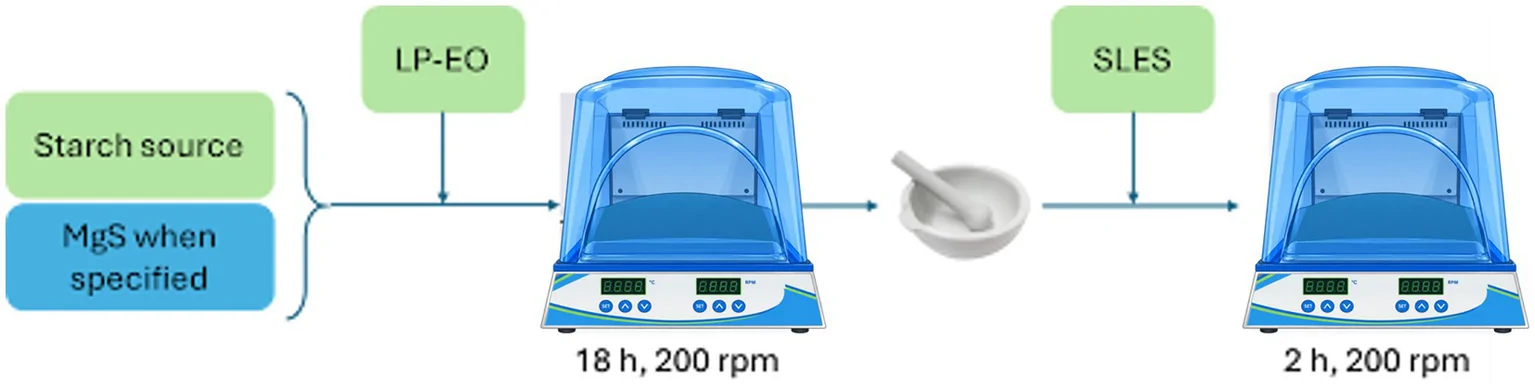
Procedure used for mixing the ingredients of the formulations for the homogeneity test. This procedure will be referred to as Method A. The same procedure was applied analogously for formulations containing CA-EO. CA-EO: *Cryptocarya alba* essential oil. LP-EO: *Laureliopsis philippiana* essential oil. MgS: Magnesium stearate. SLES: Sodium lauryl ether sulfate.

**Table 1:** Evaluation criteria for homogeneity, mixing, thermal stability, and long-term stability tests.

Score	Visual homogeneity	Absence of lumps	Color uniformity	Tactile texture	Fluidity	Agglomeration tendency
1	Heterogeneous mixture. Oil is poorly distributed.	Big and numerous lumps.	Irregularly distributed coloration, presence of clearly defined, colorful, and colorless zones.	Extremely rough or compact, hard to handle.	The mixture does not flow once the container is inclined.	Completely compacted.
2	Slightly heterogeneous. The mixture seems uniform.	Dispersed middle-sized lumps.	Slightly uniform color distribution Saturation differences are scarce.	Rough, highly cohesive particles.	Slightly fluid Requires strong stirring to move.	Persistent lumps. Visibly clumping.
3	Mostly homogeneous. Slightly visible differences in texture.	Small lumps. Easily disintegrable.	Mostly uniform color.	Soft. Lightly rough.	The mixture flows slowly without agitation when the container is inclined.	Reversible clumping when agitated.
4	Uniform. Non-visible irregular zones.	Not visible lumps. Smooth surface.	Homogeneous and stable color. Not visible color spots.	Fine consistency, homogeneous Smooth to the touch.	The mixture flows easily with a slight agitation when the container is inclined.	Slight agglomeration. The granular structure is stable.
5	Completely homogeneous, the whole sample is visually uniform.	Completely free of lumps. Uniform texture.	Completely uniform color intensity is well dispersed throughout the formulation.	Silky. Without any palpable roughness.	The mixture flows effortlessly without any agitation.	No Agglomeration, the mixture stays dispersed.

### 2.3 Mixing test

This stage aimed to identify the most efficient mixing method for maximizing the homogeneity of the formulations. Two mixing methods were evaluated, considering that the distribution of MgS within powder blends, as well as its effects on flow-related properties, may depend on blending conditions and the order of component incorporation (26, 27). Method A is described in section 2.2, Figure 2. For Method B (Figure 3), the starch source was first combined with the EO, followed by the addition of MgS and finally SLES, with an intermediate grinding step to enhance dispersion. Only the formulations prepared with MgS were subjected to the mixing test, namely, SLP1-MgS, SLP2-MgS, and SLP3-MgS. At this stage, the EO concentration was reduced to 2%, the dye concentration was halved, and the proportion of SLES was increased. Mixing was carried out in the Incu-Shaker™ Mini at 200 rpm and complemented with manual grinding when necessary to break agglomerates. The formulations were evaluated using the same organoleptic scale described in Table 1. The method that achieved the highest degree of component integration and the best powder flow was selected for preparing prototypes used in subsequent stages of this study. Final prototype selection was based on the overall balance of homogeneity, powder flow, agglomeration tendency, essential-oil incorporation, handling characteristics, and subsequent physical stability.

**Figure 3:**
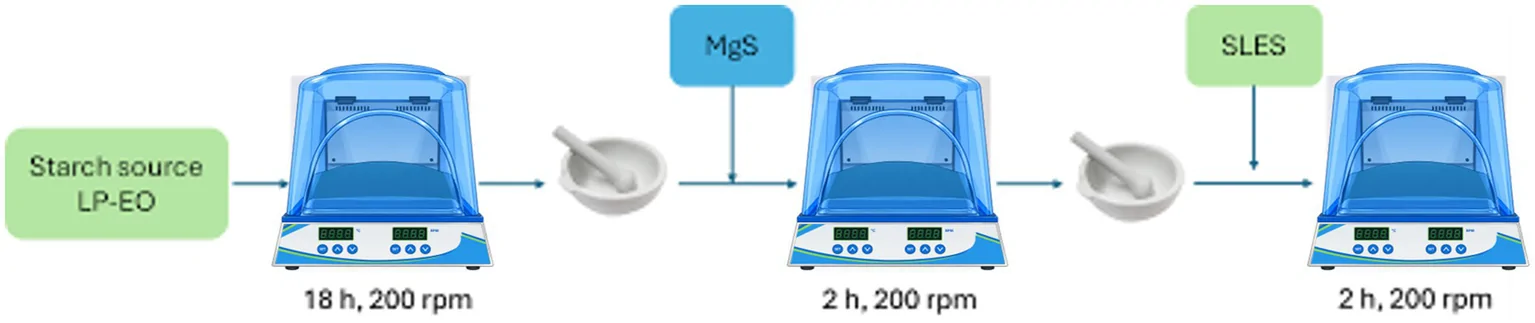
Procedure used for mixing the ingredients of the formulations for the mixing test. This procedure will be referred to as Method B. The same procedure was applied analogously for formulations containing CA-EO. CA-EO: *Cryptocarya alba* essential oil. LP-EO: *Laureliopsis philippiana* essential oil. MgS: Magnesium stearate. SLES: Sodium lauryl ether sulfate.

#### 2.3.1 Score scale

To assess component integration and overall formulation uniformity, a qualitative scoring system comprising seven physical and organoleptic parameters was applied during the homogeneity, mixing, thermal stability, and long-term stability tests. Each parameter was scored on a 1 to 5 scale according to the descriptors presented in Table 1, which were established based on evaluation criteria commonly considered in shampoo and cosmetic formulation studies (24, 28, 30). The evaluations were performed by a single, non-blinded evaluator, corresponding to the researcher conducting experimental procedures. Each sample or experimental condition was assessed once at the corresponding evaluation time point; therefore, no independent replicate organoleptic assessments were performed. Consequently, inter-evaluator concordance and intra-evaluator agreement analyses were not applicable.

### 2.4 Preliminary physicochemical characterization of the formulations

For the macroscopical physicochemical evaluation, cornstarch/MgS formulations were prepared using mixing method A, resulting in three samples: SLP (LP-carrying formulation), SCA (CA-carrying formulation), and SVE (vehicle formulation). Measurements were performed on 10 g samples. For the present exploratory formulation study, each final prototype (SLP, SCA, and SVE) was prepared as a single experimental batch. Independently prepared replicate batches were not produced; therefore, batch-to-batch reproducibility was not assessed. The tests included pH measurement, thermal stability, mechanical stability, and long-term stability evaluation (28, 29).

In addition to the macroscopic stability assessment, the final formulations were subjected to complementary physicochemical characterization. Scanning electron microscopy (SEM) was used to examine particle morphology and surface characteristics. Fourier-transform infrared spectroscopy (FTIR) was performed to compare the spectral profiles of SCA, SLP, and SVE with those of CA-EO and LP-EO, while differential scanning calorimetry (DSC) was used to evaluate the thermal behavior of the formulations and isolated essential oils.

Moisture content was determined gravimetrically from the mass loss after heating. Powder flow properties were evaluated through bulk and tapped volume measurements, from which the Hausner ratio (HR) and Carr index (CI) were calculated. HR was calculated as the ratio between tapped density and bulk density, whereas CI was calculated as the relative difference between tapped and bulk density. HR and CI measurements were performed in triplicate for each formulation, while moisture content corresponded to a single determination per formulation. Detailed SEM, FTIR, DSC, moisture, HR, and CI results are provided in the Supplementary Material.

pH was determined using rehydrated solutions prepared at a 1:10 ratio (shampoo: distilled water), mixing 0.5 g of sample with 4.5 mL of distilled water. Measurements were performed using a previously calibrated AQUASEARCHER™ AB23PH pH meter to evaluate formulation compatibility and detect potential changes associated with instability or degradation processes (28, 29). Because the formulations were developed as dry powder prototypes, pH adjustment was not performed before the biological evaluations. Modifying the pH of the selected powder system would have required reformulation and subsequent reassessment of homogeneity, powder flow, essential-oil distribution, and physical stability. Therefore, the selected prototypes were evaluated without further pH modification, while the alkaline pH was considered a formulation limitation to be addressed in subsequent optimization stages.

Thermal stability was assessed by incubating aliquots of each formulation at 50 °C for 120 h, with organoleptic evaluations conducted every 24 h, while long-term stability was evaluated through bench storage at room temperature for 9 months. In both tests, the organoleptic parameters described in Table 1 were evaluated.

Mechanical stability was assessed by centrifugation as an accelerated physical stability test, since centrifugal stress can promote particle rearrangement and reveal tendencies toward compaction, phase separation, sedimentation, or loss of powder uniformity that may not be evident under static storage conditions (29, 31). Briefly, 0.2 g aliquots were centrifuged at 13,000 rpm for 30 min at room temperature to detect signs of compaction, flocculation, or sedimentation. These results were assessed using the qualitative criteria described in Table 2 (28, 29, 31). The thermal, mechanical, and long-term assays were designed primarily to assess macroscopic and physical stability. Quantitative longitudinal chemical profiling of the essential oils during storage was not performed.

**Table 2:** Evaluation criteria for the mechanical stability test.

Score	Compaction	Flocculation	Sedimentation
1	The powder completely compacts; forms a solid and unmanageable mass	Large flocs or aggregates are formed; clearly visible clusters that do not disperse	The powder separates into distinct layers; high sedimentation is observed
2	Visible compaction of the powder; the mass can be separated after agitation	Slight flocculation; dispersed aggregates are present; clusters disperse after agitation	Moderate sedimentation, some particles remain suspended
3	No compaction is observed; the powder remains loose	No flocculation is observed; the mixture is free of visible aggregates	No sedimentation is observed; particles remain fully suspended

### 2.5 *In vitro* antibacterial evaluation of the final formulations

#### 2.5.1 Agar diffusion assay

The antibacterial activity of the final dry shampoo formulations was evaluated against *Staphylococcus pseudintermedius* ATCC 49444 and three clinical isolates (SP 1, SP 9, and SP 13). SCA, SLP, and SVE were assessed using an agar diffusion assay, with chlorhexidine 2% included as the reference antiseptic control and an untreated culture as the growth control. Inhibition zone diameters were measured in millimeters after incubation, and assays were performed in triplicate. Complete results are provided in the Supplementary Material.

#### 2.5.2 Serial dilution and minimum inhibitory concentration (MIC) assay

The final formulations were additionally evaluated using a serial dilution assay. Before testing, the dry shampoos were dispersed in Mueller–Hinton broth at a 1:3 ratio. SCA and SLP contained 3% of their respective essential oils, and the dilution series was therefore expressed as equivalent essential-oil concentrations. The evaluated concentration range was 30.72 to 0.06 μg/mL. SVE, which contained no essential oil, was evaluated at the corresponding formulation dilution levels and served as the vehicle control. Positive and negative growth controls were included in each assay.

Minimum inhibitory concentration (MIC) was determined as the lowest tested equivalent essential-oil concentration associated with absence of detectable bacterial growth under the evaluated conditions. The complete dilution data are presented in the Supplementary Material.

#### 2.5.3 Viable bacterial counts

To provide a quantitative assessment of bacterial survival, aliquots from the serial dilution assay were cultured and viable bacterial recovery was quantified as colony-forming units per milliliter (CFU/mL). CFU mL^−1^ values were calculated as the number of colonies counted × 1,000. These measurements were performed for *S. pseudintermedius* ATCC 49444 and the clinical isolates SP 1, SP 9, and SP 13. Complete CFU/mL results for each formulation and dilution level are provided in the Supplementary Material.

### 2.6 Pre-clinical safety test

Before exploratory microbiological and tolerability assessment in dogs, a preclinical safety test was performed in mice, comprising skin irritation measurements and an ex vivo histological analysis. For this purpose, eighteen healthy male and female *Mus musculus* (BALB/c) mice were obtained from the Experimental Platform of the Faculty of Dentistry, University of Chile, in April 2025, to include biological variability. The animals were divided into three groups, with 6 animals each. According to the formulation applied, groups are as follows: group treated with *C. alba* EO-loaded dry shampoo (SCA), group treated with *L. philippiana* EO-loaded dry shampoo (SLP) and group treated with the vehicle (SVE). All animals were cared for according to the Ethics Committee of the Odontology Faculty of the University of Chile (CICUA: 24814-ODO-UCH-e3). The subsequent efficacy test in dogs was conducted only after obtaining written informed consent from the owners and approval from the corresponding animal ethics committee.

All mice were housed in a conventional facility in a temperature-controlled room with a 12-h light:12-h dark cycle, at the Odontology Faculty of the University of Chile. On June 9th, all animals were weighed, and the hair from a 1 × 2 cm dorsal area was carefully removed using an electric clipper, without the use of chemical depilatory agents. The first formulation application was performed 1 hour after hair removal. Irritation, inflammation, and adverse signs were registered 1, 4, and 8 h after product application. Second doses of the formulations were administered once the 8-h observation period was completed. For days 2, 3, and 4 of the irritation test, the same application protocol was performed. On the fifth day of the irritation test, all animals were euthanized by controlled CO_2_ exposure. Immediately after, skin samples of the dorsal treated skin were collected and transferred to 15 mL centrifuge tubes with formaldehyde (10%) for histological analysis (18, 20, 22).

#### 2.6.1 Skin irritation measurements

A skin irritation test was conducted to assess the degree of skin irritation associated with shampoo application. Two criteria were used to describe irritability as detailed in Table 3.

**Table 3:** Score system used to evaluate irritation.

Score	Erythema	Edema
0	Without lesion	Without edema
1	Slightly reddened	Slight edema
2	Moderately reddened	Moderately sized edema (>1 mm elevation)
3	Severely reddened or burnt	>1 mm elevation and extended beyond treated area

#### 2.6.2 Ex-vivo histological analysis

For histological evaluation, 5 μm tissue sections were first subjected to deparaffinization with xylene (Biopack) for 15 min. The samples were then rehydrated through sequential ethanol washes of decreasing concentration (100, 95, 80, 70, and 50%), with each step lasting 5 min. Sections were stained with hematoxylin and eosin (H&E, Biopack) for 5 min, followed by removal of excess stain under running water for 2 min. Subsequently, the slides were dehydrated using ascending ethanol concentrations (50, 70, 80, 95, and 100%) and mounted with a microscopy mounting medium (Biopack). After drying, five representative microscopic fields from each experimental group were examined under bright-field microscopy using an Olympus microscope. Images were acquired with a DFC 295 digital camera (Olympus) using a 4 × objective lens and a 10 × ocular lens, corresponding to a total magnification of 40×.

Histological sections were independently evaluated by two specialized pathologists who were blinded to the experimental groups. Tissue sections were assessed according to the characteristic histopathological features established for experimental skin injury models, including epithelial integrity, epidermal thickness, epithelial cellularity, inflammatory cell infiltration, blister formation, dermal architecture, edema, vascular congestion, and other morphological alterations associated with tissue injury and repair (32–34). Histopathological findings were interpreted according to these established criteria. In cases of discrepant interpretations, the slides were jointly reviewed until consensus was reached before assigning the final histopathological classification. The histopathological assessment was qualitative, and no numerical semiquantitative grading score was prospectively assigned to the evaluated parameters.

### 2.7 Microbiological evaluation in dogs

#### 2.7.1 Study design, ethical approval, randomization, allocation concealment, and blinding

An exploratory, prospective, randomized, controlled, four-arm parallel-group clinical study was conducted in client-owned dogs clinically diagnosed with superficial bacterial pyoderma. Dogs were recruited from several veterinary care centers in Santiago, Chile, including private clinics and university-affiliated veterinary services. The study protocol was approved by the corresponding animal ethics committee under approval code ID14/2024, dated 28 June 2024. Written informed consent was obtained from all owners before enrollment.

Thirty-two dogs were allocated in a 1:1:1:1 ratio to four parallel treatment groups, with eight dogs planned per group: shampoo containing *Cryptocarya alba* essential oil (SCA), shampoo containing *Laureliopsis philippiana* essential oil (SLP), vehicle shampoo (SVE), or the chlorhexidine-based commercial comparator Inveclor®.

During the study, oclacitinib or comparable non-corticosteroid antipruritic therapies were permitted when clinically required. In contrast, corticosteroids, systemic or topical antibiotics, antiseptics, and natural products with potential antimicrobial or anti-inflammatory activity were not allowed.

Dogs were not eligible for enrollment if they had received systemic or topical antibiotics, corticosteroids of any class, or natural products with potential antimicrobial or anti-inflammatory activity within the 15 days preceding baseline evaluation. Therefore, all enrolled dogs completed a minimum 15-day treatment-free period before microbiological sampling and initiation of the assigned topical treatment.

The random allocation sequence was generated using the Datacapt Randomization platform. Simple randomization was applied at the individual-dog level, without stratification, blocking, or other restrictions. The randomization sequence and the correspondence between study codes and treatment groups were accessible only to Daniela Siel, who served as the allocation custodian and was not involved in the blinded clinical outcome assessments, clinical laboratory analyses, or microbiological evaluations.

Allocation concealment was maintained by restricting access to the randomization sequence and treatment-code list to the allocation custodian. Eligibility was confirmed, written owner consent was obtained, and each dog was enrolled and assigned an individual study identification code before the corresponding treatment allocation was accessed. The clinicians responsible for recruitment and clinical evaluation had no access to the allocation sequence and could not predict the treatment assigned to the next enrolled dog.

All study products were dispensed in identical containers labeled exclusively with the corresponding study identification code. The containers did not display the formulation name, essential oil, vehicle, or comparator identity. Although all products were provided in identical coded containers, the effectiveness of blinding was not formally assessed, and treatment identification based on potential organoleptic differences cannot be completely excluded. Dog owners, treating clinicians, and clinicians responsible for follow-up and outcome assessment were blinded to treatment allocation throughout the study.

Clinical records and biological samples were identified exclusively using coded study identification numbers. Personnel performing hematological and biochemical analyses, cytological assessments, bacterial culture, colony-count evaluation, and bacterial identification using the VITEK® system were also blinded to treatment allocation. Treatment codes were not disclosed to clinical or laboratory assessors until completion of the corresponding evaluations and analyses.

Reporting of the randomization, allocation-concealment, implementation, and blinding procedures was structured according to the PetSORT recommendations for randomized trials in client-owned dogs and cats (35).

The primary exploratory outcome of the canine study was the microbiological response at Day 21, assessed through bacterial culture, bacterial detection, and predefined bacterial-burden categories based on CFU counts. Clinical observations performed during follow-up were considered supportive assessments of local tolerability and general clinical evolution rather than independent quantitative efficacy outcomes. Because a standardized quantitative lesion-severity score was not prospectively applied and complete standardized photographic records were not available for all enrolled dogs, no retrospective clinical lesion score was generated.

Following allocation, the assigned topical product was applied by the owner directly to the affected skin areas twice daily, ideally at approximately 12-h intervals, for 21 consecutive days. The product was applied using protected hands and was left on the skin without rinsing. After each application, owners were instructed to prevent the dogs from licking, grooming, or otherwise removing the product for approximately 10–20 min. Standardized instructions were provided to the owners to promote consistent product administration. Clinical follow-up was conducted on Days 0, 7, 14, and 21.

#### 2.7.2 Microbiological sampling and bacterial identification

Microbiological specimens were collected from dogs clinically diagnosed with superficial bacterial pyoderma before treatment initiation and after completion of the 21-day topical treatment period. Samples were taken from active cutaneous lesions using sterile cotton swabs, according to standard approaches for microbiological evaluation in canine superficial pyoderma (17, 36). Prior to sampling, superficial debris was carefully removed from the lesion area with sterile gauze, without applying antiseptic agents, in order to avoid affecting bacterial recovery. The swab was gently rolled across the lesion surface to collect exudate and bacterial material, and samples were promptly transported under refrigerated conditions to the MICROVET Diagnostic Laboratory, Faculty of Veterinary and Animal Sciences, University of Chile, for further processing.

Upon arrival, samples were inoculated onto blood agar plates and incubated at 37 °C for 24–48 h. Colonies with morphological characteristics suggestive of staphylococci were selected and subcultured to obtain pure bacterial isolates. Species identification was then carried out using the VITEK® automated identification system (bioMérieux), enabling identification of *Staphylococcus pseudintermedius* at the species level (19). For each sample, the presence or absence of *S. pseudintermedius* was recorded. Detection of other *Staphylococcus* spp. was also considered to further describe the bacterial profile of the lesions. Isolates were classified as *S. pseudintermedius*, *Staphylococcus aureus*, or other *Staphylococcus* species. Antimicrobial susceptibility was evaluated using the corresponding automated susceptibility panel. Oxacillin susceptibility was used as the phenotypic indicator for the identification of methicillin-resistant *S. pseudintermedius* (MRSP), with isolates showing resistance to oxacillin considered phenotypically compatible with MRSP.

To assess the microbiological response to treatment, bacterial burden was estimated by counting colony-forming units (CFU) recovered from lesion swab cultures at baseline and after 21 days of topical dry shampoo application. Baseline cultures were considered positive when bacterial counts exceeded 300 CFU/swab, corresponding to a very high or uncountable bacterial load. After treatment, bacterial growth was categorized as absent or non-significant when 0–15 CFU/swab were detected, countable when values ranged from 16 to 300 CFU/swab, and uncountable when bacterial counts exceeded 300 CFU/swab.

### 2.8 Sample-size calculation

An *a priori* sample-size calculation was performed using G*Power version 3.1.9.6 and independently verified using R version 4.4.3 within RStudio version 2024.12.1. The expected effect size was estimated from unpublished preliminary data generated by our research group. For the overall comparison of the microbiological response among four equally allocated treatment groups, the calculation considered Cohen’s w = 0.60, a two-sided type I error rate of 0.05, and 80% statistical power. The analysis indicated that a minimum total sample size of 32 dogs was required, corresponding to eight animals per group. This sample size was also consistent with VICH GL43 recommendations applicable to target-animal safety and local tolerance evaluations of dermally administered veterinary products. The study was designed to detect large microbiological effects and common tolerability concerns and was not powered to detect small or moderate effects or to establish equivalence or non-inferiority among treatments.

### 2.9 Statistical analysis

Statistical analyses were performed using GraphPad Prism version 10 and R version 4.4.3 within RStudio version 2024.12.1. The statistical approach and revised analyses were reviewed in consultation with a biostatistician. The individual animal was considered the experimental unit. All statistical tests were two-sided, and statistical significance was established at *p* < 0.05.

pH measurements were technical replicates and were therefore summarized descriptively as mean ± standard deviation without inferential statistical comparison.

For continuous outcomes analyzed using parametric methods, model assumptions were evaluated primarily through graphical inspection of residuals, including quantile–quantile plots and residual-versus-fitted plots. Normality of model residuals was additionally assessed using the Shapiro–Wilk test, and homogeneity of variance was evaluated using the Brown–Forsythe test. Because formal normality tests have limited power in small samples, selection of the statistical approach was based on the combined assessment of graphical diagnostics, residual distribution, and variance homogeneity rather than on the Shapiro–Wilk test alone. When parametric assumptions were not adequately satisfied, appropriate non-parametric or robust alternatives were used.

Microbiological outcomes were analyzed according to their scale and data structure. Bacterial detection at Day 21 was treated as a binary categorical outcome, whereas bacterial burden was analyzed according to the predefined CFU categories. Because of the limited sample size and sparse cell counts, overall between-group comparisons at Day 21 were performed using the Fisher–Freeman–Halton exact test. Cramér’s V was calculated as an effect-size measure for the overall association between treatment group and categorical microbiological outcomes. Baseline-to-Day-21 changes in bacterial detection were evaluated using the exact McNemar test, accounting for the paired nature of repeated observations within the same dog. Only animals with microbiological results available at both time points were included in paired analyses. Changes in bacterial species identification were summarized descriptively using individual transition frequencies because several categories contained sparse or zero cell counts, precluding reliable multicategorical paired inference. Missing observations were not imputed, and the number of animals contributing to each analysis is reported.

Where secondary pairwise comparisons were performed, *p*-values were adjusted using the Holm procedure. For multiple hematological and biochemical safety outcomes, the Benjamini–Hochberg procedure was used to control the false discovery rate. Both unadjusted and adjusted *p*-values were reported where relevant, with statistical inference based on adjusted values when multiplicity correction was applicable. Missing observations were not imputed, and the number of animals contributing to each analysis is reported.

## 3 Results

### 3.1 Homogeneity test

Organoleptic parameters—visual appearance, absence of lumps, color consistency, tactile texture, fluidity, agglomeration tendency, and aroma intensity (Table 1)—were used to assess formulation uniformity and handling properties, which are key factors for the performance and acceptability of topical powders. As shown in Table 4, formulations containing MgS obtained higher general mean scores than their counterparts without MgS in the cornstarch- and tapioca-based matrices. The general mean score increased from 2.6 in SLP1 to 4.0 in SLP1-MgS, and from 3.4 in SLP2 to 4.7 in SLP2-MgS. The mixed-starch formulation also showed an increase in the general mean score after MgS incorporation, from 3.0 in SLP3 to 4.3 in SLP3-MgS. Among all formulations evaluated, SLP2-MgS showed the highest overall performance, with a general mean score of 4.7, followed by SLP3-MgS and SLP1-MgS, with mean scores of 4.3 and 4.0, respectively. Aroma intensity remained constant across all formulations, with a score of 5. Overall, the tapioca-based formulation containing MgS showed the highest mean score (4.7), compared with 2.6, 4.0, 3.4, 3.0, and 4.3 for SLP1, SLP1-MgS, SLP2, SLP3, and SLP3-MgS, respectively. Formulations containing MgS also exhibited lower agglomeration tendency and improved EO dispersion within the matrix.

**Table 4:** Organoleptic scores obtained for all formulations evaluated in the homogeneity test.

Parameter	SLP1	SLP1-MgS	SLP2	SLP2-MgS	SLP3	SLP3-MgS
Visual homogeneity	2.0	4.0	3.0	5.0	2.0	4.0
Absence of lumps	2.0	3.0	3.0	4.0	2.0	4.0
Color uniformity	2.0	5.0	3.0	5.0	3.0	5.0
Tactile texture	2.0	3.0	4.0	5.0	3.0	4.0
Fluidity	3.0	4.0	3.0	4.0	3.0	4.0
Agglomeration tendency	2.0	4.0	3.0	5.0	3.0	4.0
Aroma intensity	5.0	5.0	5.0	5.0	5.0	5.0
General mean	2.6	4.0	3.4	4.7	3.0	4.3

SLP, Shampoo with Laureliopsis philippiana essential oil.

### 3.2 Mixing test

Mixing test results are summarized in Table 5. Overall, Method A produced mixtures (SLP1-MA, SLP2-MA, and SLP3-MA) with improved flow and lower particle cohesion, while Method B (SLP1-MB, SLP2-MB, and SLP3-MB) generated fine lumps and greater agglomeration, indicating less efficient dispersion of both the solid matrix and the EO. Color uniformity did not vary between formulations and mixing methods, indicating that both methods produced an equally distributed EO in the formulation. The lump formation tendency score was lower for SLP3-MgS than for SLP1-MgS and SLP2-MgS formulations, and independent of the mixing method used.

**Table 5:** Organoleptic scores obtained for the formulations evaluated using mixing Method A and Method B in the mixing test.

Parameter	SLP1-MA	SLP1-MB	SLP2-MA	SLP2-MB	SLP3-MA	SLP3-MB
Visual homogeneity	5.0	4.0	4.0	3.0	4.0	3.0
Absence of lumps	4.0	3.0	3.0	3.0	3.0	3.0
Color uniformity	5.0	5.0	5.0	5.0	5.0	5.0
Tactile texture	4.0	4.0	4.0	3.0	4.0	4.0
Fluidity	5.0	5.0	4.0	4.0	3.0	2.0
Agglomeration tendency	4.0	3.0	4.0	3.0	2.0	2.0
Aroma intensity	3.0	3.0	3.0	3.0	3.0	3.0
General mean	4.3	3.9	3.9	3.4	3.4	3.1

SLP, shampoo with Laureliopsis philippiana essential oil, prepared through Method A and B (MA, MB).

### 3.3 Macroscopic physicochemical characterization of the optimized formulation

#### 3.3.1 pH measurements

pH measurements (Table 6) showed slight variation among SLP, SCA, and SVE. All three formulations exhibited similar alkaline values, ranging from 10.93 to 11.16, with low variability, as reflected by the small standard deviations. These results indicate that incorporation of the essential oils did not markedly alter the pH of the powder matrix. Given the alkaline nature of the formulations, their suitability for repeated topical application and potential implications for dermal tolerability are discussed further in Section 4.2.1.

**Table 6:** pH values for all formulations.

Formulation	pH
SLP	10.93 ± 0.06
SCA	11.01 ± 0.03
SVE	11.16 ± 0.03

SLP, shampoo containing Laureliopsis philippiana essential oil; SCA, shampoo containing Cryptocarya alba essential oil; SVE, vehicle shampoo.

#### 3.3.2 Thermal stability

Thermal stability results (Tables 7–9) showed gradual changes in sensory and physical properties with increasing incubation time. Visual homogeneity and color uniformity remained unchanged throughout the 120-h assay, whereas tactile texture, fluidity, agglomeration tendency, and aroma intensity showed progressive decreases in score. Both EO-containing formulations exhibited a reduction in aroma intensity during thermal exposure, consistent with loss of volatile components; however, the chemical composition of the essential oils was not quantitatively assessed during this assay. Therefore, these findings are interpreted as evidence of preliminary physical stability under the evaluated thermal conditions rather than confirmation of chemical stability.

**Table 7:** Thermal stability score for shampoo loaded with *Laureliopsis philippiana* essential oil (SLP).

Parameter	0	24	48	72	96	120
Visual homogeneity	4.0	4.0	4.0	4.0	4.0	4.0
Absence of lumps	3.5	3.0	3.0	3.0	3.0	3.0
Color uniformity	4.0	4.0	4.0	4.0	4.0	4.0
Tactile texture	4.0	3.0	3.0	2.0	2.0	2.0
Fluidity	4.0	4.0	4.0	3.5	3.0	3.0
Agglomeration tendency	3.5	3.0	3.0	3.0	2.0	2.0
Aroma intensity	4.0	2.0	2.0	2.0	2.0	2.0
General mean	3.9	3.3	3.3	3.1	2.9	2.9

**Table 8:** Thermal stability score for shampoo loaded with *Cryptocarya alba* essential oil (SCA).

Parameter	0	24	48	72	96	120
Visual homogeneity	4.0	4.0	4.0	4.0	4.0	4.0
Absence of lumps	3.5	3.0	2.5	2.5	2.0	3.0
Color uniformity	4.0	4.0	4.0	4.0	4.0	4.0
Tactile texture	4.0	4.0	3.0	2.5	2.5	2.5
Fluidity	4.0	4.0	4.0	3.7	3.0	3.0
Agglomeration tendency	3.5	3.0	3.0	3.0	2.0	2.0
Aroma intensity	3.0	2.5	2.0	2.0	2.0	2.0
General mean	3.8	3.5	3.2	3.0	2.8	2.8

**Table 9:** Thermal stability score for shampoo for SVE.

Parameter	0	24	48	72	96	120
Visual homogeneity	4.0	4.0	4.0	4.0	4.0	4.0
Absence of lumps	4.0	3.0	3.0	3.0	2.5	2.0
Color uniformity	5.0	5.0	5.0	5.0	5.0	5.0
Tactile texture	4.0	4.0	4.0	3.5	3.0	2.5
Fluidity	4.0	4.0	4.0	4.0	3.5	3.0
Agglomeration tendency	3.0	3.0	3.0	3.0	2.5	2.5
Aroma intensity	0.0	0.0	0.0	0.0	0.0	0.0
General mean	3.4	3.3	3.3	3.2	2.9	2.7

#### 3.3.3 Mechanical stability

Centrifugation data (Table 10) shows that all formulations obtained a compaction score of 2. For flocculation, SLP and SCA obtained a score of 2, whereas SVE showed the highest score, with a value of 3. For sedimentation, SLP and SVE obtained scores of 3, while SCA showed a lower score of 2. The general mean scores were 2.3 for SLP, 2 for SCA, and 2.7 for SVE, with SVE showing the highest general mean score in the mechanical stability test, followed by SLP and SCA.

**Table 10:** Score for shampoo loaded with SLP, SCA, and SVE.

Parameter	SLP	SCA	SVE
Compaction	2.0	2.0	2.0
Flocculation	2.0	2.0	3.0
Sedimentation	3.0	2.0	3.0
General mean	2.3	2.0	2.7

#### 3.3.4 Long-term stability

Long-term stability test results (Tables 11–13) showed preservation of several macroscopic properties of the three formulations tested (SLP, SCA, and SVE) during the 9-month evaluation period. Visual homogeneity and color uniformity remained unchanged throughout the 9-month evaluation period in all formulations. For SLP, the general mean score decreased from 3.9 at baseline to 3.0 at 3 months, and to 2.9 at both 6 and 9 months. For SCA, the general mean score decreased from 3.7 at baseline to 2.9 at 3 and 6 months, and to 2.7 at 9 months. For SVE, the general mean score decreased from 4.0 at baseline to 3.6 at 3 months, and to 3.4 at both 6 and 9 months. Aroma intensity decreased from 5 to 3 after 3 months in both EO-containing formulations and remained at this score until month 9, whereas SVE remained odorless throughout the assay. Scores related to tactile texture, fluidity, and agglomeration tendency showed reductions over time, while absence of lumps remained constant in SVE and decreased after 3 months in both EO-containing formulations.

**Table 11:** Long-term stability score for shampoo loaded with *Laureliopsis philippiana* essential oil (SLP).

Parameter	0 m	3 m	6 m	9 m
Visual homogeneity	4.0	4.0	4.0	4.0
Absence of lumps	3.0	2.0	2.0	2.0
Color uniformity	4.0	4.0	4.0	4.0
Tactile texture	3.0	2.0	2.0	2.0
Fluidity	4.0	3.0	2.0	2.0
Agglomeration tendency	4.0	3.0	3.0	3.0
Aroma intensity	5.0	3.0	3.0	3.0
General mean	3.9	3.0	2.9	2.9

**Table 12:** Score for SCA.

Parameter	0 m	3 m	6 m	9 m
Visual homogeneity	4.0	4.0	4.0	4.0
Absence of lumps	3.0	2.0	2.0	2.0
Color uniformity	4.0	4.0	4.0	4.0
Tactile texture	3.0	2.0	2.0	2.0
Fluidity	4.0	3.0	3.0	2.0
Agglomeration tendency	3.0	2.0	2.0	2.0
Aroma intensity	5.0	3.0	3.0	3.0
General mean	3.7	2.9	2.9	2.7

**Table 13:** Score for SVE.

Parameter	0 m	3 m	6 m	9 m
Visual homogeneity	4.0	4.0	4.0	4.0
Absence of lumps	4.0	4.0	4.0	4.0
Color uniformity	5.0	5.0	5.0	5.0
Tactile texture	5.0	4.0	4.0	4.0
Fluidity	5.0	4.0	4.0	4.0
Agglomeration tendency	5.0	4.0	3.0	3.0
Aroma intensity	0.0	0.0	0.0	0.0
General mean	4.0	3.6	3.4	3.4

Overall, visual homogeneity and color uniformity were maintained during the nine-month storage period, while gradual changes were observed in texture, fluidity, agglomeration tendency, and aroma intensity. These results indicate preservation of the macroscopic appearance of the formulations with progressive changes in powder-handling properties over time. Because no quantitative chemical analysis of the essential-oil composition was performed during storage, these observations should be interpreted as preliminary evidence of physical rather than chemical stability.

#### 3.3.5 Complementary physicochemical characterization

Additional physicochemical characterization of the final dry shampoo formulations was performed by scanning electron microscopy (SEM), Fourier-transform infrared spectroscopy (FTIR), differential scanning calorimetry (DSC), moisture determination, and powder flow measurements (Supplementary Figures S1–S3 and Supplementary Table S4). SEM examination showed a heterogeneous particulate morphology in SCA, SLP, and SVE, with granular structures and particle aggregates observed throughout the samples (Supplementary Figure S1).

FTIR analysis showed broadly similar spectral profiles among SCA, SLP, and SVE, with the formulation spectra predominantly characterized by the bands associated with the common powder matrix (Supplementary Figure S2). The isolated CA-EO and LP-EO exhibited distinct spectral features, particularly within the fingerprint region, whereas no major new absorption bands were observed in the EO-containing formulations compared with the vehicle formulation.

DSC thermograms showed a similar overall thermal profile for the three dry shampoo formulations over the evaluated temperature range (Supplementary Figure S3). SCA and SLP displayed thermal events comparable to those observed in SVE, while the isolated essential oils showed comparatively limited thermal transitions within the analyzed range. No marked additional thermal event attributable exclusively to EO incorporation was evident in the formulated powders.

Powder flow measurements showed Hausner ratios of 1.62 ± 0.02, 1.63 ± 0.08, and 1.70 ± 0.06 for SCA, SLP, and SVE, respectively. The corresponding Carr index values were 38.30 ± 0.74%, 38.39 ± 3.09%, and 40.92 ± 3.47%, respectively (Supplementary Table S4). Moisture contents were 12.61% for SCA, 14.02% for SLP, and 10.69% for SVE. Overall, SCA and SLP showed similar flow-related parameters, whereas SVE presented the highest Hausner ratio and Carr index.

### 3.4 Preliminary antimicrobial activity

As an initial assessment of the antimicrobial activity of the dry shampoo formulations, agar diffusion assays were performed against selected clinical isolates of *S. pseudintermedius* (Supplementary Table S5). Neither SCA nor SLP produced measurable inhibition zones against the three tested clinical isolates, whereas the 2% chlorhexidine control showed clear inhibition, with mean inhibition zones ranging from 19.73 to 23.85 mm. These findings indicate that the dry shampoo formulations did not exhibit detectable antimicrobial activity under the conditions of the agar diffusion assay.

As a complementary exploratory assessment, the antimicrobial activity of the dry shampoo formulations was evaluated by broth microdilution against selected *S. pseudintermedius* strains (Supplementary Table S6), together with quantitative viable bacterial counts across the corresponding dilution levels (Supplementary Tables S7–S10). In contrast to the agar diffusion results, SCA and SLP showed inhibitory activity at equivalent EO concentrations ranging from 0.12 to 0.24 μg/mL across the tested strains. The viable-count data showed lower bacterial recovery at higher formulation concentrations and increasing CFU/mL as the formulations were progressively diluted (Supplementary Tables S7–S10). However, inhibitory responses and reduced bacterial recovery were also observed with the vehicle formulation (SVE), indicating that the observed response was not restricted to the EO-containing formulations.

### 3.5 Irritation results

All measurements of skin irritation were scored at 0, showing that neither the EO-containing formulations nor the vehicle produced erythema or edema at the treated site. Serial photographic documentation (Figure 4) enabled evaluation of the cutaneous response in the shaved and demarcated dorsal area corresponding to the site of formulation application. Throughout the observation period, the animals’ skin remained intact, with no macroscopic signs of cutaneous irritation, including erythema, edema, ulceration, exudation, severe desquamation, open lesions, crust formation, bleeding, or necrotic areas. The treated area maintained a regular boundary and homogeneous coloration over time. General welfare parameters—including appearance, food and water intake, body temperature, respiratory and heart rate, and spontaneous and provoked behavior—remained within normal ranges throughout the experiment.

**Figure 4:**
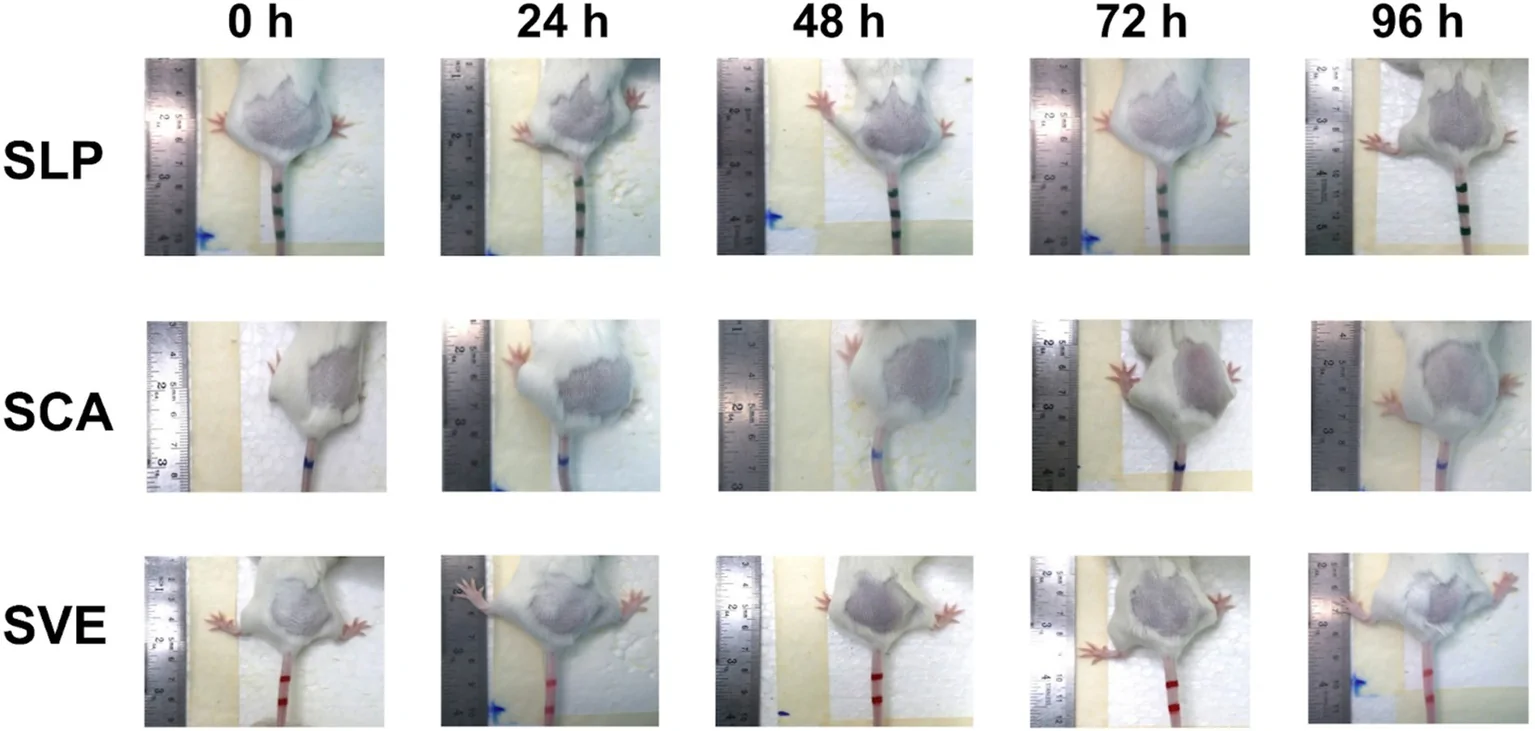
Photographic record of the shaved dorsal area of three mice (first row: Prototype containing LP-EO; second row: Prototype containing CA-EO; third row: Vehicle without EO during the safety assay), showing no evident macroscopic skin alterations.

Body weight showed minor fluctuations during the study, including both increases and decreases generally below 5%, with no consistent pattern across individuals, cages, or treatments. No progressive or sustained weight loss was observed during the follow-up period.

### 3.6 Histological analysis

Tissue samples from the dorsal skin of mice were collected to evaluate, at the cellular level, potential signs of irritation, inflammation, or tissue damage associated with the application of the formulations. Histological analysis (Figure 5) revealed that the cutaneous architecture appeared preserved, with a continuous, well-defined epidermis and no evidence of ulceration, erosion, or marked hyperplasia. The underlying dermis maintained a normal organization of collagen fibers, without apparent inflammatory infiltrates or signs of interstitial edema. Likewise, visible skin appendages exhibited preserved morphology, consistent with healthy tissue. Taken together, the comparative histological analysis demonstrates that none of the evaluated formulations induced evident histopathological alterations in the animal’s skin, with findings consistent with physiologically normal tissue.

**Figure 5:**
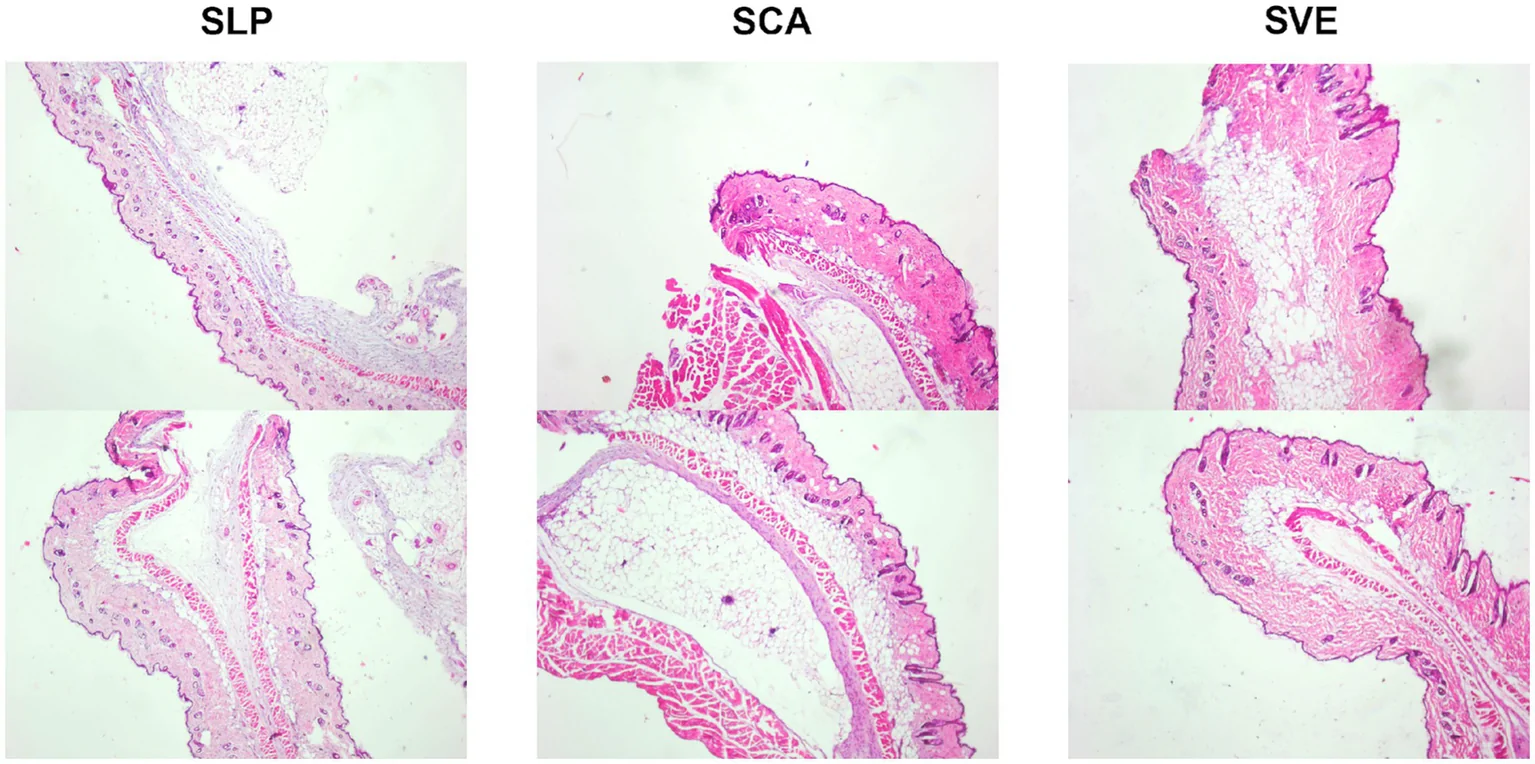
Histological samples obtained from mouse epidermis, grouped by shampoo formulation. Images were acquired by light microscopy using a 4 × objective lens and a 10 × ocular lens, corresponding to a total magnification of 40×. SLP: Shampoo with LP-EO, SCA: Shampoo with CA-EO, SVE: Vehicle shampoo without EO.

### 3.7 Microbiological evaluation in dogs

To provide preliminary evidence of antimicrobial activity in the target species, a microbiological evaluation was conducted in client-owned dogs diagnosed with superficial pyoderma. Dogs were initially allocated into four treatment groups. However, one dog from the vehicle shampoo group discontinued the study before the final microbiological evaluation; therefore, the final microbiological analysis included eight dogs in the SCA group, eight dogs in the SLP group, seven dogs in the SVE group, and eight dogs in the Inveclor® group. At baseline, all but one dog were culture-positive for *Staphylococcus* spp., with *S. pseudintermedius* being the predominant species identified.

On Day 21, *S. pseudintermedius* remained the most frequently detected species; however, changes in bacterial identification were observed in some animals, including transitions between *S. pseudintermedius* and *S. aureus*, shifts toward other staphylococcal species, and cases in which no cocci were detected in culture (Table 14). Several transitions were observed, including persistence of *S. pseudintermedius*, transitions to other staphylococcal species, and absence of detectable cocci at Day 21. Because several categories contained sparse or zero cell counts, no formal inferential comparison of multicategorical species transitions was performed.

**Table 14:** Bacterial strains identified by VITEK® between day 1 and day 21 in dogs with superficial pyoderma (*n* = 31).

Strain identified on day 1	Strain identified on day 21		
	*S. pseudintermedius*	*S. aureus*	Other spp.	No bacteria
*S. pseudintermedius*	15	5	2	8
*S. aureus*	0	0	0	0
Other spp.	1	0	0	0

The data represents the number of dogs showing each transition in bacterial species between baseline (day 1) and at the end of treatment (day 21). “Other spp.” includes *Aerococcus viridans* and *Staphylococcus warneri*. “No bacteria” indicates absence of detectable cocci in culture on day 21.

At Day 21, bacterial elimination, defined as the absence of detectable cocci in culture, was achieved in the SCA and Inveclor® groups, but not in the SVE and SLP groups.

The distribution of bacterial-load categories at Day 21 differed numerically among treatment groups but did not reach statistical significance (Fisher–Freeman–Halton exact test, *p* = 0.639; Cramér’s *V* = 0.277). The highest bacterial elimination rate was observed in the Inveclor® group, with absence of detectable cocci in 4 of 8 dogs (50.0%). Among the EO-loaded shampoo groups, bacterial elimination was observed only in the SCA group, with 3 of 8 dogs (37.5%) showing absence of detectable cocci at Day 21. In contrast, all dogs in the SLP group remained culture-positive, as did all dogs in the vehicle shampoo group (SVE), with bacterial presence detected in 8 of 8 and 7 of 7 dogs, respectively (Table 15). Although the effect-size estimate suggested a non-negligible association between treatment group and bacterial-load category, the small group sizes and sparse cell counts resulted in considerable uncertainty. These findings should therefore be interpreted as exploratory.

**Table 15:** Bacterial presence and absence on day 21 by treatment group in dogs with superficial pyoderma.

Group	*n*	Presence	Absence
SLP	8	8	0 (0.0%)
SCA	8	5	3 (37.5%)
SVE	7	7	0 (0.0%)
Inveclor®	8	4	4 (50%)

Presence indicates detection of any *Staphylococcus* species by culture and VITEK® identification on day 21. Absence indicates no detectable cocci in culture. SCA, shampoo containing *Cryptocarya alba* essential oil; SLP, shampoo containing *Laureliopsis philippiana* essential oil; SVE, vehicle shampoo.

Additionally, bacterial load was categorized at Day 21 to provide a more detailed assessment of the microbiological response. The highest proportion of dogs reaching the non-significant bacterial load category was observed in the SCA group (4/8; 50.0%), followed by the Inveclor® group (3/8; 37.5%), the SVE group (2/7; 28.6%), and the SLP group (1/8; 12.5%). Countable bacterial loads were observed in 2 of 8 dogs from the SCA group (25.0%), 1 of 8 dogs from the SLP group (12.5%), 2 of 7 dogs from the SVE group (28.6%), and 2 of 8 dogs from the Inveclor® group (25.0%). The highest proportion of dogs remaining in the uncountable category was observed in the SLP group (6/8; 75.0%), followed by the SVE group (3/7; 42.9%), the Inveclor® group (3/8; 37.5%), and the SCA group (2/8; 25.0%; Table 16). Comparison of bacterial load distribution among treatment groups using the Fisher–Freeman–Halton exact test did not reveal statistically significant differences (*p* = 0.8781).

**Table 16:** Bacterial load categorization on day 21 by treatment group. Bacterial load was categorized based on CFU counts on day 21. Non-significant: 0–15 CFU/swab; Countable: 16–300 CFU/swab; Uncountable: >300 CFU/swab.

Group	n	Non-significant	Countable	Uncountable
SLP	8	1 (12.5%)	1 (12.5%)	6 (75.0%)
SCA	8	4 (50.0%)	2 (25.0%)	2 (25.0%)
SVE	7	2 (28.6%)	2 (28.6%)	3 (42.9%)
Inveclor®	8	3 (37.5%)	2 (25.0%)	3 (37.5%)

At Day 21, bacterial growth was categorized as non-significant or significant to assess the microbiological response among treatment groups (Figure 6). The highest proportion of dogs with non-significant bacterial growth was observed in the SCA group, with 4 of 8 dogs (50.0%), followed by the Inveclor® group, with 3 of 8 dogs (37.5%). In the SVE group, non-significant growth was observed in 2 dogs, while 5 showed significant growth and 1 had no available information. In the case of the SLP group, one dog showed non-significant bacterial growth at Day 21, while significant bacterial growth was observed in the remaining cases.

**Figure 6:**
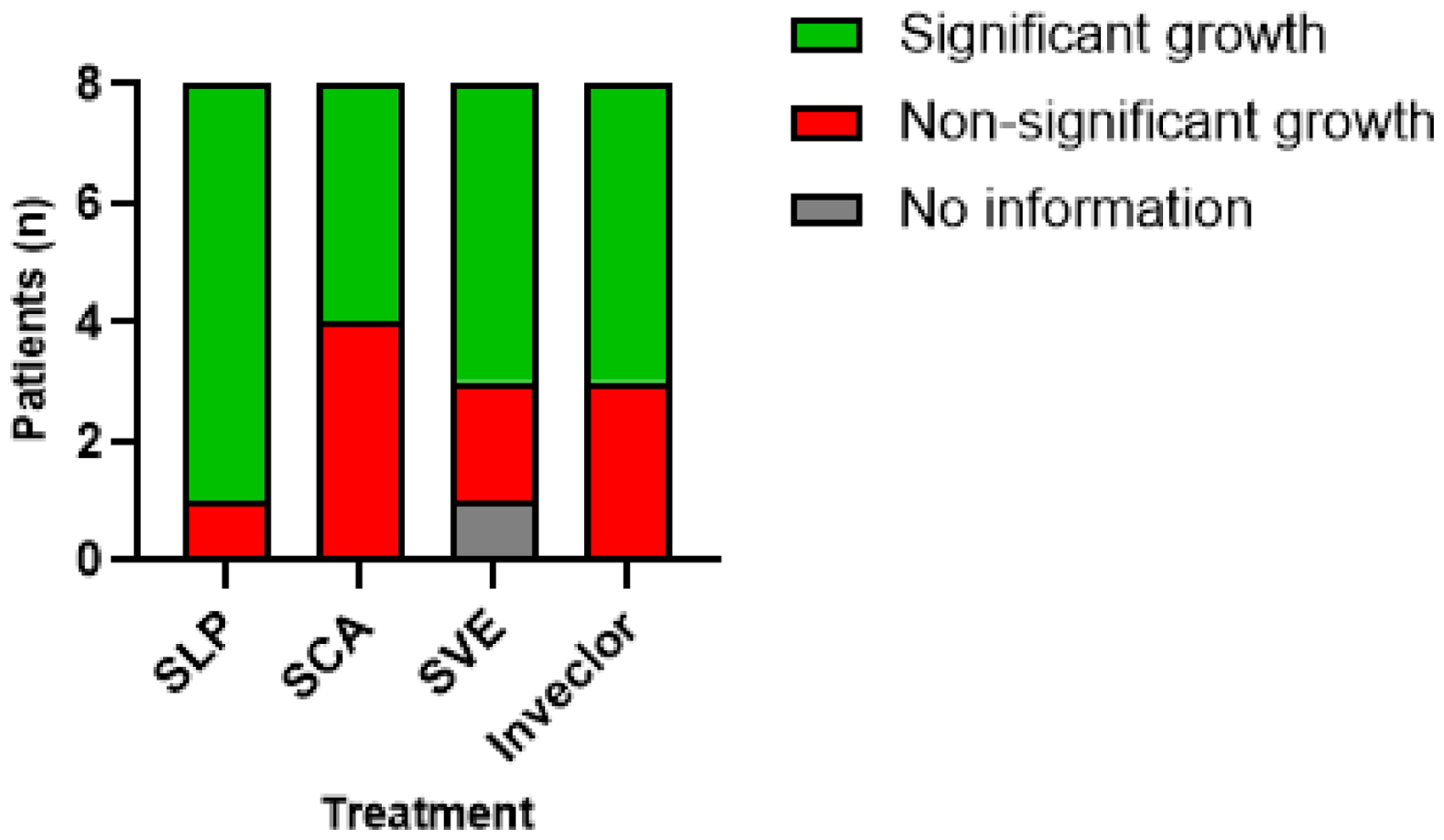
Significant growth (>15 CFU) or non-significant growth of cocci in final microbiological cultures (Day 21) across treatment groups. Fisher–Freeman–Halton exact test did not reveal significant differences among treatment groups (*p* = 0.485). Gray indicates unavailable data. SLP: Shampoo containing *L. philippiana* essential oil; SCA: Shampoo containing *C. alba* essential oil; SVE: Vehicle shampoo without antimicrobial agent.

When bacterial growth was dichotomized according to the predefined CFU threshold, no statistically significant overall difference was detected among treatment groups (Fisher–Freeman–Halton exact test (*p* = 0.485); Cramér’s *V* = 0.2). SCA showed the highest proportion of dogs reaching the lower bacterial-growth category. However, confidence intervals were wide and the estimated between-group differences remained imprecise.

Antimicrobial susceptibility testing showed that none of the *S. pseudintermedius* isolates recovered during the study were resistant to oxacillin. Therefore, no isolates phenotypically compatible with MRSP were detected.

## 4 Discussion

### 4.1 Shampoo formulation

#### 4.1.1 Homogeneity test

MgS is an established excipient in pharmaceutical and cosmetic powder systems, commonly used as a lubricant and anti-adherent to improve flow by reducing particle–particle friction and cohesion (26, 27, 37). These properties were considered relevant for the present starch-based formulations because the incorporation of a lipophilic EO may promote agglomeration, uneven distribution, and increased cohesion within the powder matrix. Consistently, formulations containing MgS (SLP1-MgS, SLP2-MgS, and SLP3-MgS) showed greater visual uniformity, better flow, and lower agglomeration tendency than their MgS-free counterparts (Table 4). These properties may favor more uniform incorporation of the EO within the powder matrix and more consistent handling during topical application (10, 38). The similar aromatic intensity observed among formulations indicates comparable organoleptic aroma intensity at the initial evaluation. Overall, these findings support the inclusion of MgS in the selected formulation for subsequent physicochemical characterization and stability testing (31, 39). Although other flow-enhancing excipients may also be suitable, this preliminary optimization stage was intended to evaluate the functional contribution of MgS rather than to perform an exhaustive screening of glidants or anti-caking agents.

#### 4.1.2 Mixing test

Efficient mixing is particularly important in powder systems containing volatile lipophilic compounds, as adequate dispersion contributes to formulation uniformity and more consistent distribution of the active components within the powder matrix. Comparing both mixing methods (Figures 2, 3), one of the key differences is the sequential order of mixing the starch source, the EO, and the MgS. Given the fact that MgS acts as an antiagglomerant, the initial mixing step of MgS and the starch source in mixing method A resulted in overall better performance in visual homogeneity and agglomeration tendency (Table 5) than method B. Aromatic intensity remained similar between samples prepared with both procedures, indicating that no marked organoleptic difference in aroma intensity was detected between the two mixing methods (Table 5) (38, 39).

When considering the combined results of the homogeneity and mixing evaluations, the cornstarch-based formulation containing MgS and prepared using Method A was selected as the base prototype for subsequent studies. Although SLP2-MgS obtained the highest individual mean score in the initial homogeneity test, the cornstarch-based formulation showed the most favorable overall balance of visual uniformity, flow behavior, agglomeration tendency, and performance under the selected mixing procedure.

An additional limitation concerns the preliminary nature of the formulation optimization process. Organoleptic parameters were evaluated by a single non-blinded evaluator, with each formulation assessed once at each corresponding time point; therefore, independent replicate assessments and inter- or intra-observer agreement analyses were not performed, potentially introducing observer-dependent bias into the selection of the optimized formulations. In addition, each final prototype was prepared as a single experimental batch, precluding assessment of batch-to-batch reproducibility. Although the complementary quantitative and analytical characterization incorporated in this study, including moisture content, Hausner ratio, Carr index, SEM, FTIR, and DSC analyses, provides additional objective information on the technological and physicochemical properties of the selected formulations (29, 31), these measurements do not eliminate the limitations associated with the initial qualitative screening process or the lack of independent batch replication. Future formulation-development studies should therefore incorporate replicated quantitative measurements, blinded assessment by multiple evaluators, objective powder-characterization parameters from the early stages of formulation selection, and independently prepared batches to confirm the reproducibility of physicochemical characteristics, powder-flow behavior, EO distribution, and stability across manufacturing runs.

### 4.2 Physicochemical characterization and stability

#### 4.2.1 pH

The alkaline values obtained are consistent with the nature of powder-based formulations intended for dry cleansing, where the presence of absorbent excipients may generate slightly basic environments. Similar behavior has been described in solid matrices incorporating EOs (10, 38). The comparable pH values observed for SLP, SCA, and SVE indicate that EO incorporation did not markedly alter this parameter.

However, all formulations exhibited alkaline pH values close to 11, which are above the ranges most commonly reported for canine skin. Canine skin pH is generally reported between 5.5 and 7.2, although broader ranges from 4.84 to 9.95 have also been described (40). In addition, commercially available non-medicated canine shampoos have been reported to exhibit pH values ranging from 5.7 ± 0.09 to 8.4 ± 0.18 (41). This difference is relevant because prolonged exposure to highly alkaline conditions may affect epidermal barrier function and potentially increase susceptibility to irritation, particularly in inflamed or compromised skin.

In the present study, pH was measured after rehydration of the dry powder at a 1:10 shampoo:water ratio and therefore does not necessarily represent the exact microenvironment generated by the dry formulation after topical application. Nevertheless, the alkaline values remain an important limitation of the current prototypes, particularly considering the reported physiological pH range of canine skin and the pH values described for commercially available canine shampoos (40, 41). pH adjustment was not performed before the biological studies because the selected formulations had already been optimized as dry powder systems, and modification of this parameter would have required reformulation followed by reassessment of homogeneity, powder flow, EO distribution, and physical stability (28, 29, 31).

Although no erythema, edema, or histological alterations were observed in the murine safety assay under the evaluated conditions, these findings do not exclude possible effects associated with repeated or prolonged application to canine skin. This consideration is relevant because topical tolerability may depend on exposure conditions and skin-barrier status, particularly in compromised or inflamed skin (18, 40). Therefore, future formulation development should include pH optimization followed by renewed physicochemical characterization and safety evaluation in the target species before extended clinical use.

#### 4.2.2 Thermal stability test

Thermal stability results (Tables 7–9) revealed gradual changes in sensory and physical properties with increasing incubation time. Visual attributes such as uniformity and color remained unchanged throughout the assay, indicating preservation of the macroscopic appearance of the formulations under the evaluated thermal conditions. Both EO-containing formulations (SLP and SCA) exhibited similar behavior, with slight decreases in powder flow and tactile texture over time, accompanied by a gradual increase in agglomeration tendency and a reduction in aromatic intensity, which is compatible with partial loss of volatile EO constituents. The essential oils incorporated into the formulations contain several volatile constituents. As previously characterized, LP-EO was predominantly composed of linalool, eucalyptol, and safrole, whereas CA-EO showed a more complex profile, with sabinene and eucalyptol among its major constituents (23). The volatile nature of these compounds may contribute to changes in aroma intensity during exposure to elevated temperatures (38). The preservation of visual parameters suggests that no major macroscopic instability occurred during thermal exposure, whereas the progressive changes in texture, agglomeration tendency, and flow indicate alterations in the physical behavior of the powders over time. During thermal incubation, SVE showed gradual reductions in fluidity and absence-of-lumps scores, indicating changes in powder handling properties over time. Overall, the results suggest that the EO-containing formulations maintained macroscopic stability broadly comparable to the vehicle, with the main changes limited to gradual variations in texture and aroma under elevated temperature conditions (31, 38).

Importantly, the thermal assay primarily assessed changes in macroscopic and physical properties. Although the decrease in aroma intensity is compatible with the loss of volatile components, the chemical composition of the essential oils was not quantitatively monitored during thermal exposure. Essential oils are complex mixtures whose constituents may undergo volatilization, oxidation, and chemical degradation depending on temperature and storage conditions (38). Therefore, oxidation, degradation, or selective volatilization of individual EO constituents cannot be excluded, and the present data should not be interpreted as demonstrating complete chemical stability. Quantitative longitudinal analysis, such as GC–MS profiling before and after thermal exposure, would be required to confirm the stability of individual EO constituents (31).

#### 4.2.3 Centrifugation stability test

The centrifugation stability test (Table 10) showed that all formulations presented the same degree of compaction after centrifugation. Differences were observed in flocculation and sedimentation: SVE showed no visible flocculation, whereas SLP and SCA exhibited slight flocculation; additionally, moderate sedimentation was observed only in SCA. Although these findings indicate some differences in mechanical behavior among formulations, no complete compaction, large aggregates, or marked phase separation were observed, suggesting that the powder systems retained their overall macroscopic integrity under the evaluated centrifugal stress (29). Overall, the results support the preliminary mechanical stability of the developed powder prototypes under the evaluated centrifugation conditions.

#### 4.2.4 Long-term stability test

The long-term stability test performed at room temperature for 9 months showed preservation of several macroscopic and physical characteristics of the SLP, SCA, and SVE formulations (Tables 11–13). Visual attributes such as color and general uniformity remained stable throughout the study, indicating preservation of the overall macroscopic appearance of the formulations. Slight decreases in texture, powder flow, and absence-of-lumps scores were observed after approximately 3 months, which may reflect progressive changes in interparticle interactions and powder cohesion during storage (29). In EO-containing formulations, aroma intensity decreased over time, which is compatible with partial loss of volatile constituents, although the characteristic aroma remained detectable. Such changes are plausible because EO components may undergo volatilization, oxidation, or degradation during storage depending on environmental and formulation conditions (38).

Overall, the observed variations were gradual and were mainly associated with powder-handling properties and aroma rather than visible loss of formulation uniformity. The consistency between the long-term and accelerated thermal observations further supports the interpretation that the prototypes retained preliminary physical stability under the evaluated conditions (29, 31). Together with the pH and centrifugation assessments, these findings provide complementary evidence of the physical behavior of the formulations under the evaluated conditions, although moderate changes in texture, flow behavior, and aroma were observed over time.

An additional limitation of the present study is that the chemical stability of the EO constituents was not quantitatively assessed during storage. Although the formulations maintained their main physical and organoleptic characteristics under the evaluated conditions, these observations cannot establish preservation of the chemical composition of the incorporated EOs. Essential-oil constituents, particularly volatile compounds, may be affected by volatilization, oxidation, or chemical degradation during storage (38). Future studies should therefore include quantitative GC–MS profiling of representative volatile constituents at baseline and after defined storage periods to characterize compositional changes and more accurately establish formulation shelf life (31).

#### 4.2.5 Complementary physicochemical characterization

The complementary physicochemical analyses provided additional objective information on the technological properties of the selected dry shampoo prototypes. SEM examination showed a heterogeneous particulate morphology in SCA, SLP, and SVE, with granular structures and particle aggregates observed across the formulations. These findings are consistent with the heterogeneous nature of multicomponent powder systems, in which differences in particle morphology, surface characteristics, and interparticle interactions may contribute to cohesion and powder-flow behavior (26, 28, 42). However, SEM observations were qualitative and should not be interpreted as a quantitative assessment of particle-size distribution or as direct evidence of the distribution of individual formulation components.

The Hausner ratio and Carr index values obtained for all three formulations were consistent with cohesive powder behavior and limited intrinsic flowability. SCA and SLP showed similar values, whereas SVE presented the highest HR and CI values. These objective measurements complement the qualitative observations obtained during formulation development and suggest that the final formulations remained cohesive powder systems and may require further optimization for reproducible handling and potential scale-up (26, 27, 29). Moisture contents ranged from 10.69 to 14.02%; however, because moisture content was determined once for each formulation, these values should be interpreted descriptively rather than as evidence of differences among formulations. Moisture is also relevant to powder performance because it can modify interparticle interactions, agglomeration, and flow behavior during storage.

FTIR analysis showed broadly similar spectral profiles among SCA, SLP, and SVE, with the spectra of the finished formulations predominantly characterized by features shared with the common powder matrix. The absence of major new absorption bands after EO incorporation suggests that no marked changes in the overall infrared profile of the formulations were detected. Nevertheless, this observation does not exclude more subtle physicochemical interactions between the EOs and excipients, and FTIR alone cannot establish chemical compatibility or stability (43).

DSC thermograms similarly showed comparable overall thermal profiles among SCA, SLP, and SVE, with no marked additional thermal event attributable exclusively to EO incorporation. These findings suggest that incorporation of CA-EO or LP-EO did not produce major detectable changes in the thermal behavior of the powder matrix within the evaluated temperature range. However, the absence of a distinct EO-related thermal event should not be interpreted as evidence that the volatile EO constituents remained chemically unchanged. FTIR and DSC can provide complementary information on spectral characteristics and thermal behavior within formulated systems (43), but they do not replace direct quantitative assessment of chemical stability. Accordingly, longitudinal chemical profiling, such as GC–MS, would still be required to establish the compositional stability of the EO constituents during storage (31, 38).

Particle-size distribution and angle of repose were not directly determined in the present study. In particular, the SEM analysis provided qualitative information on particle morphology and aggregation but should not be interpreted as a quantitative particle-size distribution measurement. Future formulation-development and scale-up studies should incorporate these additional powder-characterization parameters to provide a more comprehensive assessment of flow behavior and manufacturing reproducibility.

### 4.3 *In vitro* antibacterial activity of the final formulations

Direct evaluation of the finished dry shampoo formulations showed assay-dependent antibacterial behavior. In the agar diffusion assay, neither SCA nor SLP produced measurable inhibition zones against *S. pseudintermedius* ATCC 49444 or the three clinical isolates evaluated, whereas the 2% chlorhexidine control produced inhibition zones of approximately 20–24 mm. This contrasts with the previously reported activity of the isolated CA-EO and LP-EO against *S. pseudintermedius* (23), indicating that antibacterial activity demonstrated for an isolated EO cannot necessarily be extrapolated to the finished formulation. Incorporation into a solid matrix may influence the availability, release, and diffusion of lipophilic EO constituents, while agar diffusion itself depends strongly on the ability of antimicrobial compounds to migrate through the agar medium (10, 38, 44). Therefore, the absence of measurable inhibition zones should be interpreted in the context of the formulation and assay rather than as definitive evidence of complete absence of antibacterial activity.

In contrast, the serial dilution experiments showed differences in viable bacterial recovery as formulation concentration decreased. SCA and SLP produced absence or marked reduction of detectable bacterial growth at higher tested concentrations, followed by increasing CFU/mL at greater dilutions, supporting a concentration-dependent response under liquid-phase conditions. Dilution-based susceptibility assays provide greater direct contact between microorganisms and the tested antimicrobial system and therefore complement diffusion-based approaches (45). Importantly, SVE also reduced bacterial recovery at several corresponding formulation dilution levels. This finding indicates that the observed effect cannot be attributed exclusively to CA-EO or LP-EO and suggests that the vehicle matrix or the physicochemical conditions generated by its components contributed to bacterial growth inhibition. Accordingly, interpretation of the MIC and CFU results should consider the complete formulation rather than the EO concentration alone.

Taken together, the discrepancy between agar diffusion and serial dilution results emphasizes the influence of the experimental system on the apparent antimicrobial performance of complex topical formulations. Previous studies have demonstrated antibacterial activity of different EOs against staphylococci, including methicillin-susceptible and methicillin-resistant *S. pseudintermedius* isolates (46, 47), but the present results suggest that incorporation into a dry powder matrix may modify the antimicrobial behavior observed with the isolated oils. Thus, the current findings provide preliminary evidence of antibacterial activity under specific *in vitro* conditions, rather than definitive confirmation of antimicrobial efficacy of the finished shampoos. Further studies should evaluate a larger collection of clinical isolates and incorporate complementary approaches such as formulation-release studies, time-kill assays, and antibiofilm models to distinguish more clearly the contributions of the EO and vehicle matrix to the observed antibacterial response.

### 4.4 Safety test in murine model

Following macroscopic physicochemical characterization, the three final formulations (SLP, SCA, and SVE) were subjected to *in vivo* testing to assess their preliminary dermal safety. A murine model was used for this purpose.

#### 4.4.1 Pre-clinical safety assessment

Photographic monitoring of representative animals during the five-day experimental period (Figure 4) allowed evaluation of the dermal response at the application site. Throughout the study, the skin remained intact with no visible signs of irritation, including erythema, edema, ulceration, exudate, severe desquamation, open lesions, crusts, bleeding, or necrosis. Minor variations in skin coloration observed among individuals were not consistent or progressive and were therefore attributed to normal physiological differences or handling during the experimental procedure (18, 20). General welfare parameters—including appearance, food and water intake, body temperature, respiratory and heart rate, and spontaneous behavior—remained within normal ranges throughout the observation period. Body weight fluctuations were minimal, generally remaining below 5%, and showed no consistent trends among animals or treatment groups, which is compatible with normal physiological variation in young animals adapting to experimental conditions (17, 19).

#### 4.4.2 Histological analysis

Histological examination of skin samples collected at the end of the trial revealed preserved tissue architecture across the SLP, SCA, and SVE groups (Figure 5), with a continuous epidermis and well-defined dermal layers. No signs of ulceration, erosion, necrosis, or hyperplasia were observed. The dermis displayed normal collagen fiber organization without evidence of inflammatory infiltration or edema, with no histological evidence of treatment-associated cellular damage or inflammatory responses under the evaluated conditions. Comparable histological features were observed in all groups, including the vehicle formulation, suggesting that the shampoo base itself did not produce detectable tissue alterations under the evaluated conditions. In particular, the EO-containing formulations (SLP and SCA) exhibited tissue responses similar to the vehicle, suggesting that EO incorporation did not introduce histological changes under the tested conditions. Together with the clinical observations, these findings provide preliminary evidence that the developed prototypes were well tolerated under the evaluated topical exposure conditions and support further dermal safety evaluation for potential veterinary application (18, 20, 48).

The preclinical dermal safety evaluation in BALB/c mice provided additional evidence supporting the tolerability of the formulations. Repeated topical applications did not produce overt adverse clinical signs, behavioral changes, erythema, edema, or histological evidence of inflammation or tissue damage under the evaluated conditions. This finding is relevant because EOs may exert biological effects in mammalian cells depending on their chemical composition and concentration; therefore, safety assessment is an essential step in the development of EO-based topical products (49). However, the results obtained in mice should be considered as preliminary preclinical evidence and do not replace safety evaluation in canine patients under clinical conditions.

An additional limitation of the present study is that dermal permeation and systemic exposure to EO constituents were not directly assessed; consequently, the extent of transdermal absorption following topical application cannot be established from the current findings. Although local dermal tolerability and general safety parameters were monitored, these assessments cannot substitute for dedicated permeation or pharmacokinetic studies. Future investigations should therefore include *in vitro/ex vivo* permeation experiments using excised canine skin and diffusion-cell systems following the principles of OECD Test Guideline 428, allowing quantitative determination of representative EO constituents retained within the skin and those reaching the receptor compartment, thereby providing information on dermal retention and transdermal absorption. Assessment of skin barrier integrity before and after exposure should also be incorporated to determine whether repeated application affects cutaneous barrier function, while complementary quantification of representative EO constituents in plasma following repeated topical administration would help characterize systemic exposure and bioavailability. Together, these approaches will be important to determine whether exposure remains predominantly local and to more comprehensively characterize the safety profile of the formulations under repeated topical use.

### 4.5 Microbiological evaluation in dogs

The microbiological assessment in dogs offered an initial *in vivo* approximation of the antimicrobial behavior of the dry shampoo formulations under clinical conditions. To characterize the response more comprehensively, three outcomes were considered: bacterial presence or absence, bacterial load categories, and classification of growth as significant or non-significant. Before treatment, nearly all dogs yielded positive cultures for *Staphylococcus* spp., supporting the infectious nature of the lesions and agreeing with the recognized predominance of *S. pseudintermedius* in canine superficial pyoderma (2, 50).

By Day 21, absence of detectable cocci in culture was recorded in the SCA and Inveclor® groups, whereas all evaluable dogs in the SLP and SVE groups remained culture positive. The highest clearance frequency was observed in the Inveclor® group, which is expected considering the established role of chlorhexidine-based topical products in the treatment of canine superficial pyoderma (2, 51). Among the essential-oil-loaded dry shampoos, SCA was the only EO-containing formulation in which culture negativity was observed, suggesting a more favorable culture-based response for the *C. alba* EO formulation.

Bacterial load categorization allowed the response to be interpreted with greater resolution than culture positivity alone. On Day 21, the SCA group had the largest proportion of dogs classified within the non-significant bacterial load category, followed by Inveclor®, SVE, and SLP. In the case of the SLP group, one dog showed non-significant bacterial growth at Day 21, while significant bacterial growth was observed in the remaining cases. In addition, SLP showed the highest proportion of dogs in the uncountable category, which suggests that the antibacterial activity previously observed *in vitro* for *L. philippiana* EO was not clearly reproduced in the culture-based *in vivo* evaluation.

The categorization of bacterial growth as significant or non-significant supported this trend, with SCA showing the highest proportion of dogs with non-significant bacterial growth at Day 21. However, comparisons among treatment groups did not reveal statistically significant differences. Therefore, these results should be interpreted as exploratory trends rather than definitive evidence of treatment efficacy. Nevertheless, bacterial load and growth categorization may represent useful endpoints for future studies, as they allow the microbiological response to be evaluated beyond a binary presence/absence outcome.

This distinction is clinically relevant because the management of canine superficial pyoderma does not depend exclusively on complete bacterial eradication. Reduction of bacterial burden, limitation of lesion progression, improvement of the skin microenvironment, and support of barrier recovery are also important therapeutic goals. Current recommendations emphasize diagnosis supported by cytology, bacterial culture when indicated, topical therapy for surface and superficial infections, and responsible antimicrobial use to reduce unnecessary systemic antibiotic exposure (2, 6). Within this framework, a topical product that helps reduce bacterial burden may be valuable even when full microbiological clearance is not achieved in every patient.

The comparatively better microbiological profile observed for SCA may be associated with the chemical composition of *C. alba* EO. This oil contains monoterpenes such as sabinene, eucalyptol, terpinen-4-ol, α-pinene, and β-pinene, which have been related to antibacterial effects involving membrane disruption, increased cell permeability, leakage of intracellular components, and interference with bacterial adhesion or persistence (10, 14, 52). Comparable mechanisms have been reported for other essential oils tested against staphylococci, including *S. pseudintermedius* isolates associated with canine pyoderma (46, 47).

The differences observed between the assay-dependent *in vitro* performance of the finished formulations and the microbiological responses obtained *in vivo* further illustrate the difficulty of extrapolating laboratory antimicrobial results to clinical settings. In dogs, bacterial dynamics are shaped by lesion characteristics, skin barrier condition, local inflammation, host immunity, grooming behavior, environmental exposure, owner adherence, and retention of the formulation on the skin. These variables are especially relevant in superficial pyoderma, which commonly occurs secondary to barrier dysfunction, allergic disease, or recurrent dermatological disorders (50, 53). For this reason, the present dog trial should be regarded as exploratory and hypothesis-generating rather than definitive.

These results should also be interpreted in the broader context of antimicrobial resistance. *S. pseudintermedius* is a key canine pathogen, and methicillin-resistant or multidrug-resistant strains remain a major concern in veterinary medicine (1, 54, 55). In the present study, none of the recovered *S. pseudintermedius* isolates showed phenotypic resistance to oxacillin. However, this finding should be interpreted cautiously, as methicillin resistance was assessed phenotypically and was not confirmed by molecular detection of resistance determinants such as *mecA*. Thus, the results indicate the absence of phenotypically detected MRSP rather than the definitive exclusion of methicillin resistance. Therefore, the development of well-tolerated topical alternatives may contribute to One Health-oriented strategies aimed at reducing reliance on systemic antimicrobials, particularly considering the potential zoonotic circulation of *S. pseudintermedius* (3, 56).

Several limitations should be acknowledged. Although the antimicrobial activity of the final dry shampoo formulations was directly evaluated using agar diffusion, serial dilution, MIC assessment, and quantitative viable bacterial counts, these analyses were preliminary and included only *S. pseudintermedius* ATCC 49444 and three clinical isolates. Therefore, the observed antibacterial responses cannot yet be considered representative of the broader bacterial population associated with canine superficial pyoderma (46, 47). Moreover, because SVE also reduced bacterial recovery at several corresponding formulation dilution levels, the contribution of the vehicle matrix cannot be excluded and the observed *in vitro* activity cannot be attributed exclusively to the incorporated EOs. The clinical study was designed to detect large microbiological effects but was not powered to detect small or moderate treatment effects or to establish definitive clinical efficacy, equivalence, or non-inferiority. In addition, microbiological outcomes were not integrated with a standardized quantitative lesion-severity score, and the individual contribution or potential synergistic interactions of the EO constituents were not experimentally determined.

Additional limitations are related to the clinical heterogeneity of the enrolled canine population. Dogs were recruited under real-world conditions from multiple veterinary centers, and a substantial proportion were mixed-breed animals. Although eligibility criteria required a minimum 15-day treatment-free period for systemic or topical antibiotics, corticosteroids, and natural products with potential antimicrobial or anti-inflammatory activity, complete standardized information regarding breed or breed type and dermatological treatments received before this exclusion period was not available for all animals. Consequently, these variables could not be incorporated into treatment-group stratification or statistical adjustment and may have contributed to variability in microbiological response. Furthermore, clinical follow-up was limited to the 21-day treatment period; therefore, persistence of the microbiological response, recurrence of superficial pyoderma, and longer-term tolerability after treatment discontinuation could not be determined.

Future studies should therefore include a larger and more diverse collection of clinical isolates, complementary time-kill, antibiofilm, formulation-release, and skin-retention studies, and larger randomized controlled clinical trials using standardized clinical outcome measures (2, 44, 50).

Overall, although the antibacterial performance of the finished formulations was assay-dependent and the between-group microbiological differences observed *in vivo* were not statistically significant, the incorporation of native plant EOs into a dry shampoo platform with preliminary physical stability and favorable preclinical tolerability represents a relevant formulation-development step. Among the EO-containing formulations, SCA showed the most favorable exploratory microbiological profile, with the highest proportion of dogs reaching a non-significant bacterial burden and the only cases of culture negativity observed among the EO-loaded shampoo groups. These findings support further investigation of *C. alba* EO-containing dry shampoos as potential topical candidates for canine superficial pyoderma, while emphasizing that larger controlled studies are required before conclusions regarding clinical efficacy can be established (2, 23).

## 5 Conclusion

This study reports the development and characterization of starch-based dry shampoo prototypes incorporating EOs from *L. philippiana* and *C. alba*, together with the assessment of their *in vitro* antibacterial performance, preclinical dermal tolerability, and exploratory microbiological response in dogs with naturally occurring superficial pyoderma associated with *S. pseudintermedius*. Overall, the different experimental stages addressed the central hypothesis that these EOs could be incorporated into dry shampoo formulations with preliminary physical stability, acceptable local dermal tolerability under the evaluated conditions, and measurable microbiological responses. The use of native Chilean plants as sources of bioactive compounds represents a potential strategy for the development of complementary topical approaches in veterinary dermatology.

From a formulation perspective, incorporation of magnesium stearate and selection of an appropriate mixing protocol improved powder homogeneity, reduced agglomeration, and enhanced handling and flow-related characteristics. Complementary physicochemical characterization further showed that the selected prototypes remained cohesive powder systems and exhibited preliminary physical stability under the evaluated conditions. However, the alkaline pH, absence of batch-to-batch reproducibility assessment, and lack of longitudinal chemical profiling remain relevant formulation limitations.

Direct *in vitro* evaluation of the finished formulations revealed assay-dependent antibacterial behavior. No measurable inhibition zones were observed by agar diffusion, whereas serial dilution and viable-count experiments showed concentration-dependent reductions in bacterial recovery. Because inhibitory effects were also observed with the vehicle formulation, the antibacterial response cannot be attributed exclusively to the incorporated EOs. These findings highlight the importance of evaluating the complete formulation rather than extrapolating antimicrobial activity from the isolated EOs alone.

The preclinical safety assessment showed that repeated topical application of the EO-containing formulations and vehicle in BALB/c mice did not produce visible erythema, edema, relevant behavioral or body-weight alterations, or histological evidence of treatment-associated inflammation or tissue damage under the evaluated conditions. These findings provide preliminary evidence of local dermal tolerability but do not replace safety assessment in the target species or dedicated studies of dermal permeation and systemic exposure.

In dogs with superficial pyoderma, numerical differences were observed among treatment groups in bacterial detection, bacterial-load categories, and significant versus non-significant growth. These differences were not statistically significant and should not be interpreted as evidence of clinical efficacy. Nevertheless, among the EO-containing formulations, SCA showed the most favorable exploratory microbiological profile, including the highest proportion of dogs reaching a non-significant bacterial burden and the only cases of culture negativity observed among the EO-loaded shampoo groups.

The leave-on dry-powder format may be particularly advantageous for localized lesions, intertriginous areas, or dogs in which repeated bathing is difficult or impractical, potentially simplifying repeated topical administration and reducing handling requirements. However, these practical advantages were not formally evaluated in the present study and should be confirmed in future usability and owner-adherence studies.

Taken together, these findings support the feasibility of incorporating *L. philippiana* and *C. alba* EOs into starch-based dry shampoo prototypes and provide preliminary evidence of physical stability, preclinical dermal tolerability, and assay-dependent antibacterial and exploratory microbiological performance. The leave-on powder format may offer practical advantages, including localized application and administration without rinsing, although these potential benefits were not formally evaluated. Further work should include pH optimization, independently prepared formulation batches, quantitative longitudinal chemical stability profiling, formulation-release, time-kill and antibiofilm studies, dermal permeation and skin-retention assessment, larger randomized controlled clinical trials incorporating validated clinical scoring systems, and extended safety evaluation before conclusions regarding clinical efficacy or long-term veterinary use can be established.

## 6 Patents

The national registration application for the antibacterial dry shampoo has been filed with INAPI Chile and is currently under review for acceptance.

## Data Availability

The original contributions presented in this study are included in the article/Supplementary materials. Further inquiries can be directed to the corresponding author.

## Footnotes

1 AGRANA STARCH. Non-aerosol Dry Shampoo with MAISITA 9040 and TAPIOCA NATURAL. Available at: https://www.agrana.com/downloadcenter?tx_thagranadownloadcenter_downloadcenter%5Baction%5D=download&tx_thagranadownloadcenter_downloadcenter%5Bcontroller%5D=Downloadcenter&tx_thagranadownloadcenter_downloadcenter%5Bdownload%5D=4856&type=97687851&cHash=6f68fc458981b7bde90b514c1f5f7bcf

2 AkzoNobel Surface Chemistry. Zephyrous Dry Shampoo. Available at: https://www.essentialingredients.com/pdf/AkzoNobel%20Zephyrous%20Dry%20Shampoo%20%282906-100%29.pdf
